# Comprehensive blood metabolomics profiling of Parkinson’s disease reveals coordinated alterations in xanthine metabolism

**DOI:** 10.1038/s41531-024-00671-9

**Published:** 2024-03-19

**Authors:** Elisa Gómez de Lope, Rebecca Ting Jiin Loo, Armin Rauschenberger, Muhammad Ali, Lukas Pavelka, Tainá M. Marques, Clarissa P. C. Gomes, Rejko Krüger, Enrico Glaab, On behalf of the NCER-PD Consortium, Geeta Acharya, Gloria Aguayo, Myriam Alexandre, Muhammad Ali, Wim Ammerlann, Giuseppe Arena, Rudi Balling, Michele Bassis, Roxane Batutu, Katy Beaumont, Regina Becker, Camille Bellora, Guy Berchem, Daniela Berg, Alexandre Bisdorff, Ibrahim Boussaad, David Bouvier, Kathrin Brockmann, Jessica Calmes, Lorieza Castillo, Gessica Contesotto, Nancy De Bremaeker, Nico Diederich, Rene Dondelinger, Nancy E. Ramia, Daniela Esteves, Guy Fagherazzi, Jean-Yves Ferrand, Katrin Frauenknecht, Manon Gantenbein, Thomas Gasser, Piotr Gawron, Soumyabrata Ghosh, Marijus Giraitis, Enrico Glaab, Martine Goergen, Elisa Gómez De Lope, Jérôme Graas, Mariella Graziano, Valentin Groues, Anne Grünewald, Wei Gu, Gaël Hammot, Anne-Marie Hanff, Linda Hansen, Michael Heneka, Estelle Henry, Sylvia Herbrink, Sascha Herzinger, Michael Heymann, Michele Hu, Alexander Hundt, Nadine Jacoby, Jacek Jaroslaw Lebioda, Yohan Jarosz, Sonja Jónsdóttir, Quentin Klopfenstein, Jochen Klucken, Rejko Krüger, Pauline Lambert, Zied Landoulsi, Roseline Lentz, Inga Liepelt, Robert Liszka, Laura Longhino, Victoria Lorentz, Paula Cristina Lupu, Tainá M. Marques, Clare Mackay, Walter Maetzler, Katrin Marcus, Guilherme Marques, Patricia Martins Conde, Patrick May, Deborah Mcintyre, Chouaib Mediouni, Francoise Meisch, Myriam Menster, Maura Minelli, Michel Mittelbronn, Brit Mollenhauer, Friedrich Mühlschlegel, Romain Nati, Ulf Nehrbass, Sarah Nickels, Beatrice Nicolai, Jean-Paul Nicolay, Fozia Noor, Marek Ostaszewski, Clarissa P. C. Gomes, Sinthuja Pachchek, Claire Pauly, Laure Pauly, Lukas Pavelka, Magali Perquin, Rosalina Ramos Lima, Armin Rauschenberger, Rajesh Rawal, Dheeraj Reddy Bobbili, Kirsten Roomp, Eduardo Rosales, Isabel Rosety, Estelle Sandt, Stefano Sapienza, Venkata Satagopam, Margaux Schmitt, Sabine Schmitz, Reinhard Schneider, Jens Schwamborn, Raquel Severino, Amir Sharify, Ekaterina Soboleva, Kate Sokolowska, Hermann Thien, Elodie Thiry, Rebecca Ting Jiin Loo, Christophe Trefois, Johanna Trouet, Olena Tsurkalenko, Michel Vaillant, Mesele Valenti, Gilles Van Cutsem, Carlos Vega, Liliana Vilas Boas, Maharshi Vyas, Richard Wade-Martins, Paul Wilmes, Evi Wollscheid-Lengeling, Gelani Zelimkhanov

**Affiliations:** 1 Biomedical Data Science, Luxembourg Centre for Systems Biomedicine (LCSB), University of Luxembourg, Esch-sur-Alzette, Luxembourg; 2 Parkinson’s Research Clinic, Centre Hospitalier de Luxembourg (CHL), Luxembourg, Luxembourg; 3 Transversal Translational Medicine, Luxembourg Institute of Health (LIH), Strassen, Luxembourg; 4 Translational Neuroscience, Luxembourg Centre for Systems Biomedicine (LCSB), University of Luxembourg, Esch-sur-Alzette, Luxembourg; 5 Luxembourg Institute of Health, Strassen, Luxembourg; 6 Luxembourg Centre for Systems Biomedicine, University of Luxembourg, Esch-sur-Alzette, Luxembourg; 7 Centre Hospitalier de Luxembourg, Strassen, Luxembourg; 8 Center of Neurology and Hertie Institute for Clinical Brain Research, Department of Neurodegenerative Diseases, University Hospital Tübingen, Tübingen, Germany; 9 Centre Hospitalier Emile Mayrisch, Esch-sur-Alzette, Luxembourg; 10 Laboratoire National de Santé, Dudelange, Luxembourg; 11 Association of Physiotherapists in Parkinson’s Disease Europe, Esch-sur-Alzette, Luxembourg; 12 Faculty of Science, Technology and Medicine, University of Luxembourg, Esch-sur-Alzette, Luxembourg; 13 Department of Epidemiology, CAPHRI School for Public Health and Primary Care, Maastricht University Medical Centre, Maastricht, the Netherlands; 14 Centre Hospitalier du Nord, Ettelbrück, Luxembourg; 15 Oxford Parkinson’s Disease Centre, Nuffield Department of Clinical Neurosciences, University of Oxford, Oxford, UK; 16 Private practice, Ettelbruck, Luxembourg; 17 Parkinson Luxembourg Association, Leudelange, Luxembourg; 18 Westpfalz-Klinikum GmbH, Kaiserslautern, Germany; 19 Oxford Centre for Human Brain Activity, Wellcome Centre for Integrative Neuroimaging, Department of Psychiatry, University of Oxford, Oxford, UK; 20 Department of Neurology, University Medical Center Schleswig-Holstein, Kiel, Germany; 21 Ruhr-University of Bochum, Bochum, Germany; 22 Luxembourg Center of Neuropathology, Dudelange, Luxembourg; 23 Department of Life Sciences and Medicine, University of Luxembourg, Esch-sur-Alzette, Luxembourg; 24 Paracelsus-Elena-Klinik, Kassel, Germany; 25 Private practice, Luxembourg, Luxembourg; 26 Oxford Parkinson’s Disease Centre, Department of Physiology, Anatomy and Genetics, University of Oxford, South Parks Road, Oxford, UK

**Keywords:** Computational biology and bioinformatics, Systems biology, Parkinson's disease

## Abstract

Parkinson’s disease (PD) is a highly heterogeneous disorder influenced by several environmental and genetic factors. Effective disease-modifying therapies and robust early-stage biomarkers are still lacking, and an improved understanding of the molecular changes in PD could help to reveal new diagnostic markers and pharmaceutical targets. Here, we report results from a cohort-wide blood plasma metabolic profiling of PD patients and controls in the Luxembourg Parkinson’s Study to detect disease-associated alterations at the level of systemic cellular process and network alterations. We identified statistically significant changes in both individual metabolite levels and global pathway activities in PD vs. controls and significant correlations with motor impairment scores. As a primary observation when investigating shared molecular sub-network alterations, we detect pronounced and coordinated increased metabolite abundances in xanthine metabolism in de novo patients, which are consistent with previous PD case/control transcriptomics data from an independent cohort in terms of known enzyme-metabolite network relationships. From the integrated metabolomics and transcriptomics network analysis, the enzyme hypoxanthine phosphoribosyltransferase 1 (HPRT1) is determined as a potential key regulator controlling the shared changes in xanthine metabolism and linking them to a mechanism that may contribute to pathological loss of cellular adenosine triphosphate (ATP) in PD. Overall, the investigations revealed significant PD-associated metabolome alterations, including pronounced changes in xanthine metabolism that are mechanistically congruent with alterations observed in independent transcriptomics data. The enzyme HPRT1 may merit further investigation as a main regulator of these network alterations and as a potential therapeutic target to address downstream molecular pathology in PD.

## Introduction

The diagnosis of Parkinson’s disease (PD) is typically based on clinical symptoms, such as tremors, rigidity, and bradykinesia, following standard guidelines for previously validated clinical assessments. While clinical diagnosis has improved continuously over the past decades and includes indicators for differential and early diagnosis, many of the covered symptoms are nonspecific. These may overlap with other neurological disorders or only show up in later stages of the disease, often resulting in a misdiagnosis or delayed diagnosis^1^. One of the main challenges in the clinical diagnosis is a long pre-motor phase of the disease, during which many common symptoms may not yet be present or may be subclinical. Differentiating PD from other neurological disorders, such as atypical or secondary parkinsonism, during this pre-motor phase can be challenging^2^. In addition, PD phenotypes can vary substantially, highlighting a need for objective biological biomarker signatures rather than diagnosis based solely on a subjective judgment.

Molecular signatures have the potential to provide more specific, accurate, and cost-effective indicators of a complex disorder such as PD. While clinical indicators mostly rely on assessing broad categories of symptomatic and disease-associated changes, molecular markers may reveal more granular pathological changes occurring already in the pre-symptomatic disease stages. By facilitating an earlier, more reliable, and specific diagnosis, molecular signatures may enable more patient-tailored and effective treatments.

In recent years, omics technologies have significantly contributed to discovering PD biomarkers. Numerous genetic, protein, and metabolic changes associated with PD have been identified through these approaches, and new insights into the disease pathogenesis were gained^3^. For instance, several genetic variants have been linked to an increased risk of developing PD^4^. Additionally, proteomic studies have revealed altered protein levels in PD brain tissue, including an increased abundance of alpha-synuclein, which plays a central role in Lewy body and Lewy neurites formation as a hallmark pathological feature of PD^5^. Finally, prior metabolomic studies in PD have identified changes in disease-relevant cellular pathways, particularly in those related to energy metabolism^6^.

Despite these advances, omics-based biomarker discovery for PD is still hampered by several limitations and challenges. While high cross-validated accuracies for PD diagnosis have been reported for some of the published omics signatures^7,8^, the training and test set sizes used to evaluate the corresponding machine learning (ML) models are often small. In addition, the signatures for the most predictive models have often been derived from tissues or body fluids with limited practical accessibility, e.g., cerebrospinal fluid (CSF), which requires a lumbar puncture for sample collection. A further common limitation of the multidimensional patterns in PD molecular biomarker signatures is that they can emerge as black-box models that are not fully intuitive to interpret^9^. Considering this and the limited sample sizes in many prior studies, more research is needed to find robust and interpretable PD-specific molecular signatures.

To contribute to the ongoing research efforts in this field, we have conducted a cohort-wide blood plasma metabolic profiling of 549 PD patients and 590 controls in the Luxembourg Parkinson’s Study (LuxPARK^10^) combined with subsequent statistical and bioinformatics pathway and network analyses. As a distinctive characteristic of other studies focusing on PD patients who have already received dopaminergic medication, we included biospecimens from all 56 untreated de novo patients available in the cohort. This subset of samples was used to distinguish between treatment-associated and treatment-independent metabolite changes.

To mechanistically interpret PD-associated alterations in the context of cellular networks and exploit prior information from complementary omics data, we have mapped the metabolomics statistics onto a dedicated genome-scale enzyme-metabolite network together with transcriptomics data from an independent PD case/control study. Through the integrated analysis of these omics data, we identified coordinated sub-network alterations, particularly in xanthine metabolism, which displayed regulatory consistent changes between metabolite abundances and the expression of enzyme-encoding genes. These consistent sub-network changes may help to pave the way towards more robust blood-based biomarker signatures and provide new insights into coordinated, disease-associated cellular process alterations in PD.

## Results

**Fig. 1:**
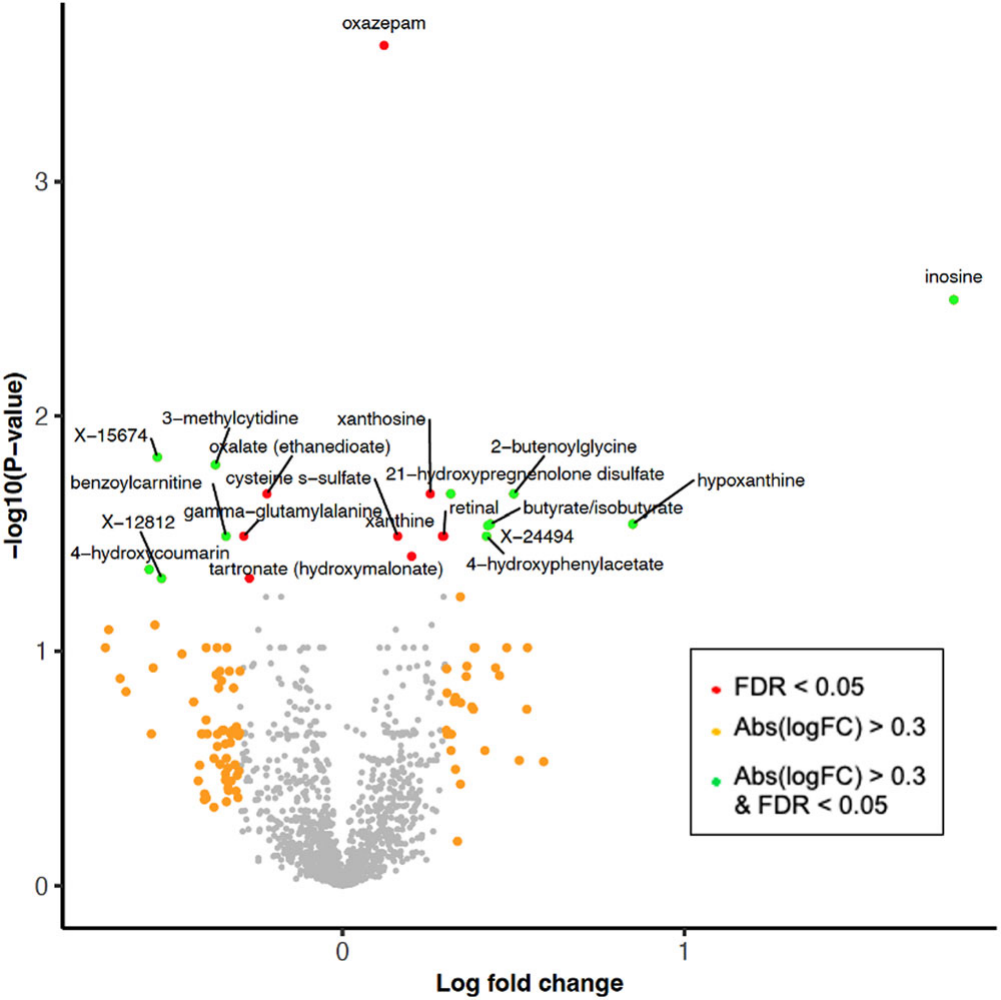
Volcano plot for the differentially abundant metabolites. Volcano plot for the differentially abundant metabolites when comparing de novo PD vs. control blood plasma samples. Metabolites displaying abundance changes with high effect size (absolute log. fold-change effect size (abs(logFC)) > 0.3) and high significance (adjusted *p* value <= 0.05) are highlighted in green, metabolites with only a high effect size are shown in orange, and metabolites with only a high significance in red.

When studying metabolite abundance changes in de novo PD patients compared to controls and in all PD patients (including subjects who had received dopaminergic treatments) vs. controls, we identified several metabolites with a statistically significant alteration (adjusted *p* value <= 0.05). Figure 1 presents a volcano plot for the de novo PD vs. control comparison, highlighting the metabolites with both high statistical significance and pronounced effect sizes. Table 1 shows the top 25 most significant metabolites in de novo PD vs. controls, and Table 2 shows the metabolites with shared significance in de novo PD vs. controls and all PD vs. controls (complete ranking tables of all significant metabolites for the individual comparisons are provided in Supplementary Tables 5 and 6; rankings for treated patients only are provided in Supplementary Table 7). The metabolites with shared significance also display the same direction of the change, i.e., the signs of the log fold-changes are identical. We grouped the significantly altered metabolites by shared functional categories to discuss them in the context of the prior literature on molecular mechanisms in PD.

**Table 1:** Top 25 most significantly differentially abundant blood plasma metabolites

Metabolite	LogFC	*P* value	Adj. *P* value
Oxazepam (sedative, likely medication effect)	**0.12**	1.83E-07	2.63E-04
Inosine^*^	**1.79**	4.44E-06	3.19E-03
X-15674	−0.54	3.12E-05	1.49E-02
3-methylcytidine	−0.37	4.49E-05	1.61E-02
2-butenoylglycine	**0.50**	8.18E-05	2.14E-02
Xanthosine^*^	**0.26**	1.17E-04	2.14E-02
Oxalate (ethanedioate)	−0.22	1.19E-04	2.14E-02
21-hydroxypregnenolone disulfate	**0.32**	1.19E-04	2.14E-02
Butyrate/isobutyrate	**0.43**	1.86E-04	2.87E-02
Hypoxanthine^*^	**0.85**	2.00E-04	2.87E-02
X-24494	**0.43**	2.23E-04	2.91E-02
Gamma-glutamylalanine	−0.29	3.23E-04	3.23E-02
4-hydroxyphenylacetate	**0.42**	3.59E-04	3.23E-02
Benzoylcarnitine	−0.34	3.64E-04	3.23E-02
Retinal	**0.30**	3.73E-04	3.23E-02
Cysteine s-sulfate	**0.16**	3.79E-04	3.23E-02
Xanthine^*^	**0.29**	3.82E-04	3.23E-02
2-hydroxy-4-(methylthio)butanoic acid	**0.20**	4.93E-04	3.94E-02
4-hydroxycoumarin (anticoagulant medication)	−0.57	5.92E-04	4.48E-02
Tartronate (hydroxymalonate)	−0.27	7.12E-04	4.89E-02
X-12812	−0.53	7.14E-04	4.89E-02
Heptanoate (7:0)	**0.34**	9.33E-04	5.86E-02
3-carboxy-4-methyl-5-pentyl-2-furanpropionate (3-CMPFP)	−0.18	9.41E-04	5.86E-02
3-hydroxybutyroylglycine	**0.30**	9.79E-04	5.86E-02
Gamma-glutamylcitrulline	−0.22	1.02E-03	5.87E-02

The top 25 most significantly differentially abundant blood plasma metabolites in the de novo PD vs. control comparison. Shown is the metabolite name (column 1), the log fold-change between de novo PD vs. control (column 2), the nominal *p* value significance (*P* value) and the adjusted *p* value (adj. *P* value). Compounds reflecting likely medication effects for PD comorbidities have been indicated in brackets behind the metabolite name. Metabolites involved in xanthine metabolism have been highlighted by the star symbol (*). In addition to the column headers highlighted in bold, the log fold-changes are shown in bold for metabolites with increased abundance in de novo PD, and in regular font for metabolites with decreased abundance.

**Table 2:** Shared significant blood plasma metabolites

Metabolite	LogFC (DPD)	*P* value (DPD)	Adj. *P* value (DPD)	LogFC (APD)	*P* value (APD)	Adj. *P* value (APD)
Inosine^*^	**1.79**	4.44E-06	3.19E-03	**0.63**	3.57E-03	3.71E-02
Hypoxanthine^*^	**0.85**	2.00E-04	2.87E-02	**0.45**	2.26E-04	5.92E-03
Gamma-glutamylalanine	−0.29	0.000323	0.032301	−0.13	4.71E-03	4.44E-02
Benzoylcarnitine	−0.34	3.64E-04	3.23E-02	−0.25	9.15E-06	7.76E-04
Retinal	**0.30**	3.73E-04	3.23E-02	**0.13**	3.30E-03	3.48E-02
4-hydroxycoumarin	−0.57	5.92E-04	4.48E-02	−0.30	1.10E-03	1.62E-02
Tartronate (hydroxymalonate)	−0.27	7.12E-04	4.89E-02	−0.18	3.45E-04	7.65E-03
X-12812	−0.53	7.14E-04	4.89E-02	−0.28	1.28E-03	1.80E-02

Shared significant blood plasma metabolites in the comparisons of de novo PD (DPD) patients vs. controls (first three columns) and all PD (APD) patients vs. controls (adjusted for dopaminergic treatment; last three columns). The columns show the metabolite names (Metabolite), the log fold-change between patients vs. controls (logFC), the nominal *p* value significance (*P* value) and the adjusted *p* value (adj. *P* value). Metabolites involved in xanthine metabolism have been highlighted by the star symbol (*). In addition to the column headers highlighted in bold, the log fold-changes are shown in bold for metabolites with increased abundance in PD, and in regular font for metabolites with decreased abundance.

### Xanthine metabolites (inosine, xanthosine, xanthine, hypoxanthine)

Among the significant abundance alterations, xanthine metabolites stand out with a shared increased abundance in de novo PD vs. control for four representatives of this group: inosine, xanthosine, xanthine, and hypoxanthine (highlighted by the star symbol in the first column of Table 1). While these changes are highly significant in de novo patients, they are not observed when comparing only treated PD patients vs. controls after multiple hypothesis testing adjustments (see Supplementary Table 7). Since the effect of dopaminergic medication on blood metabolite levels in the group of treated patients cannot be removed entirely by the conducted filtering and statistical adjustments, a higher measurement variation in this group as compared to the de novo PD subgroup is in line with our expectations. However, the difference between treatment-naïve and treated patients may also reflect the later disease stage of the latter group (see Table 3).

**Table 3:** Tabular overview of baseline subject characteristics

	PD	De novo PD	Controls	*P* value (PD vs. controls)	*P* value (de novo PD vs. controls)
***N*** **(female/male)**	549 (189/360)	56 (14/42)	590 (206/384)	0.9	0.14
**Age at assessment**	66.0 ± 10.7	67.2 ± 11.4	61.7 ± 11.7	2.2E-10	8.2E-4
**MDS-UPDRS III**	32.8 ± 14.6	32.3 ± 14.2	3.69 ± 5.0	< 2.2E-16	< 2.2E-16
**H&Y scale**	2.1 ± 0.7	1.8 ± 0.57	–	–	–
**Disease duration since initial symptom**	12.3 ± 7.3	6.68 ± 5.4	–	–	–
**BMI (kg/m2)**	27.5 ± 4.8	28.2 ± 4.8	27.6 ± 4.8	0.67	0.4
**MoCA**	25.4 ± 3.2	24.9 ± 3.3	26.9 ± 2.6	< 2.2E-16	4.7E-7
**SCOPA-AUT**	14.4 ± 8.0	8.9 ± 5.3	7.3 ± 5.6	< 2.2E-16	0.052
**Diabetes (yes/no)**	46/503 8.4%/91.6%	4/52 7.1%/92.9%	48/542 8.1%/91.9%	0.91	1.0
**Hypercholesterol-emia (yes/no)**	210/339 38.3%/61.7%	27/29 48.2%/51.8%	219/371 37.1%/62.9%	0.71	0.11
**History of cancer (yes/no)**	60/489 10.9%/89.1%	4/52 7.1%/92.9%	49/541 8.3%/91.7%	0.16	1.0

Tabular overview of baseline subject characteristics for Parkinson’s disease (PD) patients and controls from the Luxembourg Parkinson’s Study (*MDS-UPDRS* Movement Disorder Society-Unified Parkinson’s Disease Rating Scale, *H&Y scale* Hoehn and Yahr scale, *BMI* body mass index, *MoCA* Montreal Cognitive Assessment, *SCOPA-AUT* Scales for Outcomes in Parkinson’s disease-Autonomic). The study groups (PD = all PD patients, de novo PD = PD patients who have not yet been treated with dopaminergic medication; Controls = healthy control subjects) and the compared characteristics are both highlighted in bold.

Interestingly, xanthine metabolites have already been implicated in PD through multiple mechanisms. One of the proposed functional links to molecular hallmarks of PD is the generation of reactive oxygen species by xanthine oxidase (also known as xanthine dehydrogenase or XDH). When XDH catalyzes the conversion of hypoxanthine to xanthine, the reactive oxygen species (ROS) O_2_^-^ and H_2_O_2_ are generated^11^. ROS can induce oxidative stress, which may damage cellular components such as mitochondria and dopaminergic neurons. In PD, this can contribute to the loss of dopaminergic neurons in the *substantia nigra* as one of the main pathological characteristics of the disease. Indeed, increased levels of XDH in the blood of PD patients have previously been reported^12^, matching our observed metabolite changes in this pathway. Changes in oxidative stress signaling in PD are further evidenced by the significant PD-associated decreased abundance of gamma-glutamylalanine (see Table 2), which is involved in the metabolism of glutathione, an antioxidant that is essential for cellular protection against oxidative damage^13^.

In addition to this association with ROS generation, xanthine and some of its derivatives, including paraxanthine, theophylline, and caffeine, have been linked to neuroprotective mechanisms in PD. In an MPTP (1-methyl-4-phenyl-1,2,3,6-tetrahydropyridine) mouse model of PD, Xu et al. demonstrated that the administration of paraxanthine, theophylline, and caffeine significantly attenuated MPTP-induced dopamine depletion, as reported in their study^14^. For caffeine in particular, several epidemiological studies have also consistently shown a significant negative correlation between its consumption and a PD diagnosis^15^, although this may be explained by inverse causation (e.g., reduced caffeine consumption due its effects as central nervous system stimulant, potentially worsening PD symptoms). Furthermore, the substituted xanthine molecule has been used as a scaffold to synthesize new drug-like compounds as a non-dopaminergic strategy for neuroprotection^16^. The main proposed mechanisms for the protective actions of xanthines and caffeine in this context include the antagonism of the adenosine A2A receptor (ADORA2A) and inhibition of monoamine oxidase type B (MAO-B). Indeed, A2A is targeted by the drug istradefylline, a xanthine derivative with a particularly long half-life, which has been approved by the Food and Drug Administration (FDA) as an add-on treatment to levodopa (L-DOPA) for Parkinson’s patients with motor fluctuations^17^. For the second proposed xanthine target, MAO-B, pharmacological inhibitors belong to the first drugs developed for treating PD^18^.

In summary, multiple different pathways have been proposed to link alterations in xanthine metabolism with pathological or protective mechanisms in PD. To better understand the specific processes involved in xanthine abundance alterations in PD and identify the potential enzymes involved, we have further investigated xanthine metabolism as part of a joint network analysis of the metabolomics data and independent PD case/control transcriptomics data (see section on “Integrative network analysis of metabolomic and transcriptomic changes in PD”).

### Catecholamine metabolism (retinal, ALDH1A1)

Besides the xanthines, the carotenoid retinal was the only other metabolite exhibiting a significantly higher abundance in both de novo PD vs. controls and all PD patients vs. controls. Retinal is the oxidized form of retinol, most well-known as a constituent of visual pigments. Interestingly, the enzyme aldehyde dehydrogenase 1A1 (ALDH1A1), responsible for converting retinal into retinoic acid, has already been implicated in the catecholaldehyde hypothesis of PD^19,20^. This hypothesis proposes that long-term increased build-up of DOPAL (3,4-Dihydroxyphenylacetaldehyde), a toxic catecholaldehyde metabolite of dopamine which is converted by *ALDH1A1* into its non-toxic form DOPAC (3,4-Dihydroxyphenylacetic acid), plays a pathogenic role in the development of the disease. It suggests that DOPAL contributes to the damage to neurons in the *substantia nigra pars compacta*, resulting in the typical motor symptoms associated with PD. Consistent with the increased levels of retinal in PD, we detected a matching PD-associated decrease in the gene expression of *ALDH1A1* in the studied PD case/control transcriptomics data (adjusted *p* value = 5.65E-04), which could result in reduced conversion of retinal to retinoic acid.

### Metabolites associated with nutrition and the microbiome (oxalate, tartronate, 4-hydroxyphenylacetate, 3-CMPFP, 3-methylcytidine)

The identified metabolites with significant PD-associations include multiple compounds previously linked to microbiome composition or diet. Among these compounds, oxalate is a naturally occurring substance present in large quantities in many plants but only found in very low concentrations in animal tissues^21^. It may therefore serve as an indicator for a predominantly plant-based diet. It displayed a significantly decreased abundance in de novo PD vs. controls (adj. *p* value = 0.021), whereas in the all-PD patients vs. control comparison, the decrease was only close to the significance threshold (adj. *p* value = 0.051). As previous studies suggested that adherence to plant-based diets, which are typically high in antioxidants and anti-inflammatory compounds, may be associated with a reduced risk of developing PD^22^ or improved motor performance^23^ and slower disease progression^24^, oxalate may warrant further study as marker for a plant-based diet and potential associated beneficial effects.

A further metabolite with known dietary associations is tartronate, which displayed a significantly reduced abundance in both all PD patients vs. controls (adj. *p* value = 7.65E-03) and de novo PD vs. controls (adj. *p* value = 0.049). Tartronate is a monosaccharide that has been detected in several natural foods, including sourdough and ground cherries, among others^25^, and linked with the presence of various bacterial species in the human microbiome^26^. While potential mechanisms linking tartronate to PD are still unknown, significant decreased serological abundances in early-stage PD have already been reported for an independent cohort for both tartronate and oxalate^27^, matching with the findings of our study.

Among the diet-associated metabolites with increased abundance, 4-hydroxyphenylacetate (4-HP) displayed significance specific only to the de novo PD vs. control comparison (adj. *p* value = 0.032). 4-HP can be found naturally in human tissues and biofluids and in several natural foods, and it is also produced by multiple microbial species^25^. However, since 4-HP does not provide a sufficiently specific marker for individual food items or bacterial species, further research is needed to link the observed increase in de novo PD to specific mechanisms.

Next, the metabolite 3-carboxy-4-methyl-5-pentyl-2-furanpropionate (3-CMPFP), which showed a significantly decreased abundance in all PD patients compared to controls (adj. *p* value = 1.34E-04) and a nominally significant decrease in de novo PD vs. control (*p* value = 9.41E-04; adj. *p* value = 5.86E-02), is a furan fatty acid previously suggested as a marker for fish oil intake^28^, which could indicate a diet with high levels of omega-3 fatty acids. The metabolite was also reported to act as a protein-bound uremic toxin and interact with reactive oxygen species (ROS), resulting in cellular damage^28^. Thus, the decrease of 3-CMPFP in PD may be linked to disease-relevant processes through indirect dietary associations or direct mechanistic pathways.

Finally, the metabolite 3-methylcytidine (m^3^C), which was significantly decreased in de novo PD vs. controls (adj. *p* value = 0.016; with a similar trend in the all PD vs. control comparison, but no statistical significance, adj. *p* value = 0.18), is a pyrimidine nucleoside previously proposed as a urinary biomarker of whole grain intake^29^. In tRNA molecules, m^3^C is a frequently observed epigenetic modification^30^ and a lack of m^3^C_32_ modifications in tRNAs has been shown to impair cytoplasmic and mitochondrial translation^31^. Furthermore, significant differences in m^3^C modifications have been reported in prefrontal lobe cortex samples of Alzheimer’s disease patients^32^, and further studies suggested functional links between defects in tRNA modifications and neurological disease^33^.

### Metabolites involved in fatty acid metabolism and β-oxidation (benzoylcarnitine, butyrate, 2-butenoylglycine)

Among the significant metabolites, we identified multiple compounds involved in fatty acid metabolism, particularly in the β-oxidation pathway. One of the most pronounced changes was observed for an acylcarnitine, benzoylcarnitine, showing a shared decreased abundance in de novo PD vs. controls and all PD patients vs. controls (see Table 2). Alterations in acylcarnitine metabolism have been associated with a dysregulation of mitochondrial β-oxidation in PD^34^. β-oxidation breaks down fatty acids into acetyl CoA (coenzyme A), which can then enter the TCA (tricarboxylic acid) cycle to generate ATP, fulfilling cellular energy needs. Acylcarnitines, such as benzoylcarnitine, are used as carriers to transport activated long-chain fatty acids into the mitochondria for β-oxidation. In PD, increased long-chain fatty acids and decreased long-chain acylcarnitines have been observed, suggesting an impairment of mitochondrial β-oxidation^34^. Interestingly, the drug zonisamide, which has been used to treat resting tremor and motor fluctuations in PD, was previously shown to increase the abundance of multiple long-chain acylcarnitines associated with improved fatty acid β-oxidation^35^.

A further metabolite associated with fatty acid β-oxidation is butyrate, a short-chain fatty acid formed by bacterial fermentation of carbohydrates, e.g., from dietary fiber, in the intestine^25^. Butyrate is found ubiquitously in plant oils and animal fat and is contained in many dairy food products^25^. Here, a significant increase in butyrate, or, respectively, its isomer isobutyrate, was observed in de novo PD vs. control but not when comparing treated PD to controls. Differences in butyrate levels may result from changes in its production or utilization. For example, differences in diet or medicine intake can influence gut bacterial butyrate production, and the gut microbiome in PD patients has been reported to have a reduced fraction of butyrate-producing bacteria in multiple studies^36–42^. Similar to the acylcarnitines, butyrate is involved in fatty acid ß-oxidation, where it serves as an intermediate metabolite. Its increased abundance may therefore indicate dysfunctional β-oxidation in PD. However, butyrate has also been proposed to influence PD symptoms through a variety of other mechanisms. For instance, sodium butyrate intake was reported to reduce PD-related motor symptoms via mechanisms associated with gut microbial dysbiosis regulation^43^, intestinal barrier protection through the activation of G-protein-coupled receptor 109 A (GPR109A)^44^, and stimulation of glucagon-like peptide-1^45^. In contrast to these proposed protective effects, a study in a mouse model of neurodegeneration using the toxin MPTP reported worsening effects of sodium butyrate administration on motor function, associated with upregulation of pro-inflammatory cytokine expression and increased colonic inflammation^46^. Overall, the potential effects of butyrate on gut dysfunction and inflammation warrant further investigation.

Finally, as a further change associated with fatty acid ß-oxidation, 2-butenoylglycine was significantly increased in de novo PD vs. controls. This metabolite belongs to the class of acylglycines, which can be produced through glycine conjugation of acyl-CoA esters. Glycine conjugation in mammals is often used as a detoxification method to promote the excretion of carboxylic acids, and the increase of crotonylglycine may therefore reflect the response to a pathological accumulation of crotonyl-CoA. Similar to butyrate, crotonyl-CoA is an intermediate in fatty acid ß-oxidation, and the alterations in crotonylglycine match with previously reported changes in fatty acid β-oxidation in PD^47^. Moreover, in anaerobic bacteria, crotonyl-CoA serves as an intermediate for butyrate production^48^, and the shared increase in crotonylglycine and butyrate may therefore reflect the same pathway alteration.

In summary, multiple observed metabolite abundance changes point to PD-associated alterations in fatty acid metabolism, specifically in fatty acid β-oxidation, where a dominant increase of intermediate metabolites matches previous independent reports of incomplete β-oxidation in PD^34,47^.

### Metabolites associated with non-dopaminergic medication

While the comparison of de novo patients vs. controls reveals PD-related metabolite changes that are independent of dopaminergic treatments, de novo patients may still take other non-dopaminergic medications that can be detected in the metabolomics profile. We therefore investigated the metabolite changes in de novo patients vs. controls for potential effects of non-dopaminergic drugs and identified two significant drug-related metabolites: oxazepam and 4-hydroxycoumarin (see Table 1). Oxazepam is the active ingredient in many sedatives and is used to treat anxiety and depression, which commonly occurs in PD^49^. While sufficiently detailed medication data for participants in the cohort was not available to confirm the intake of corresponding drugs, treatment with sedatives is the most plausible explanation for the significantly increased oxazepam abundance observed in de novo PD. By contrast, 4-hydroxycoumarin was significantly reduced in de novo PD. This compound serves as an anticoagulant for conditions caused by a blood clot^50^. However, coumarins also have anti-inflammatory, antioxidant and neuroprotective actions^51^ with potential relevance for PD. In particular, coumarins can inhibit monoamine oxidase (MAO) enzymes^52^, which are well-established PD drug targets (see the section above on the actions of xanthines on MAO-B). Thus, potential protective actions of 4-hydroxycoumarin resulting in a preferential detection of this compound in controls may merit further study.

### Altered metabolites with unknown identity

Among the top 25 most significant metabolites in the de novo PD vs. controls comparison, the chemical identity could not be resolved for one metabolite with an increased abundance in PD (metabolite ID: X-24494) and two metabolites with a decreased abundance (metabolite IDs: X-15674, X-12812; the latter also showed a significant decrease in all PD patients vs. controls). Possible reasons for this include the lack of a reference standard for these molecules or the chemical interference of other metabolites. As the libraries and annotations by the metabolomics service provider Metabolon and the reference databases are continuously updated, we will complement the current annotations with every significant update in the future and publish relevant new findings on the GitLab repository associated with this study (see section “Data availability”).

### Pathway enrichment analysis of metabolite changes in PD

While the analysis of individual metabolites already revealed multiple significant PD-associated changes in metabolites with similar functions, we performed further complementary pathway analyses to identify and interpret coordinated alterations in the data. For this purpose, we tested the over-representation of differentially abundant metabolites in de novo PD vs. controls in pathways from the KEGG (Kyoto Encyclopedia of Genes and Genomes) database^53^ (RRID:SCR_012773) and metabolite sets representing chemical structure classes in the software MetaboAnalyst^54^ (RRID:SCR_015539), using the entire set of named metabolites as background reference (see Supplementary Table 8) and focusing on the metabolite sets with at least 5 metabolites.

**Fig. 2:**
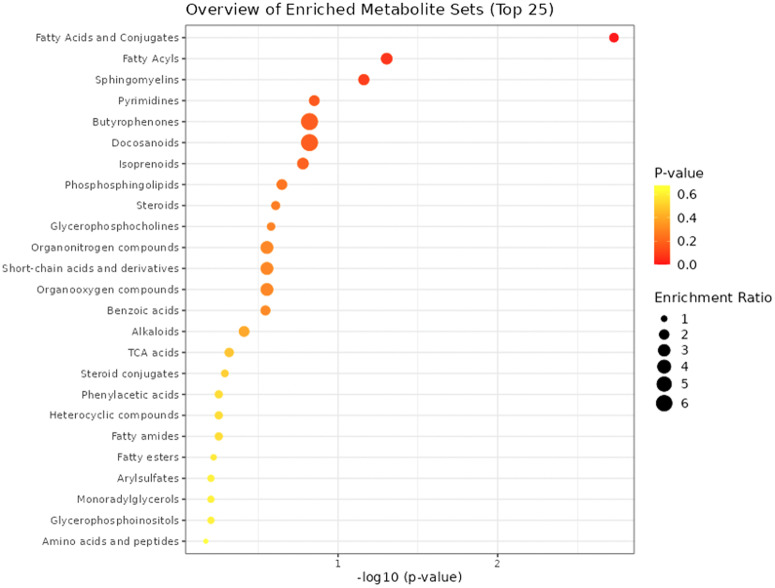
Metabolite set enrichment analysis results. Metabolite set enrichment analysis results for the de novo PD vs. control comparison using chemical structure classes (main set) in the software MetaboAnalyst. The horizontal axis shows the negative decadic logarithm of the *p* value, and the vertical axis shows the pathways, sorted by decreasing significance from the top. The color gradient from red to yellow reflects increasing *p* values, and the size of the dots reflects the effect size for each metabolite set (enrichment ratio; see the legend on the right).

**Fig. 3:**
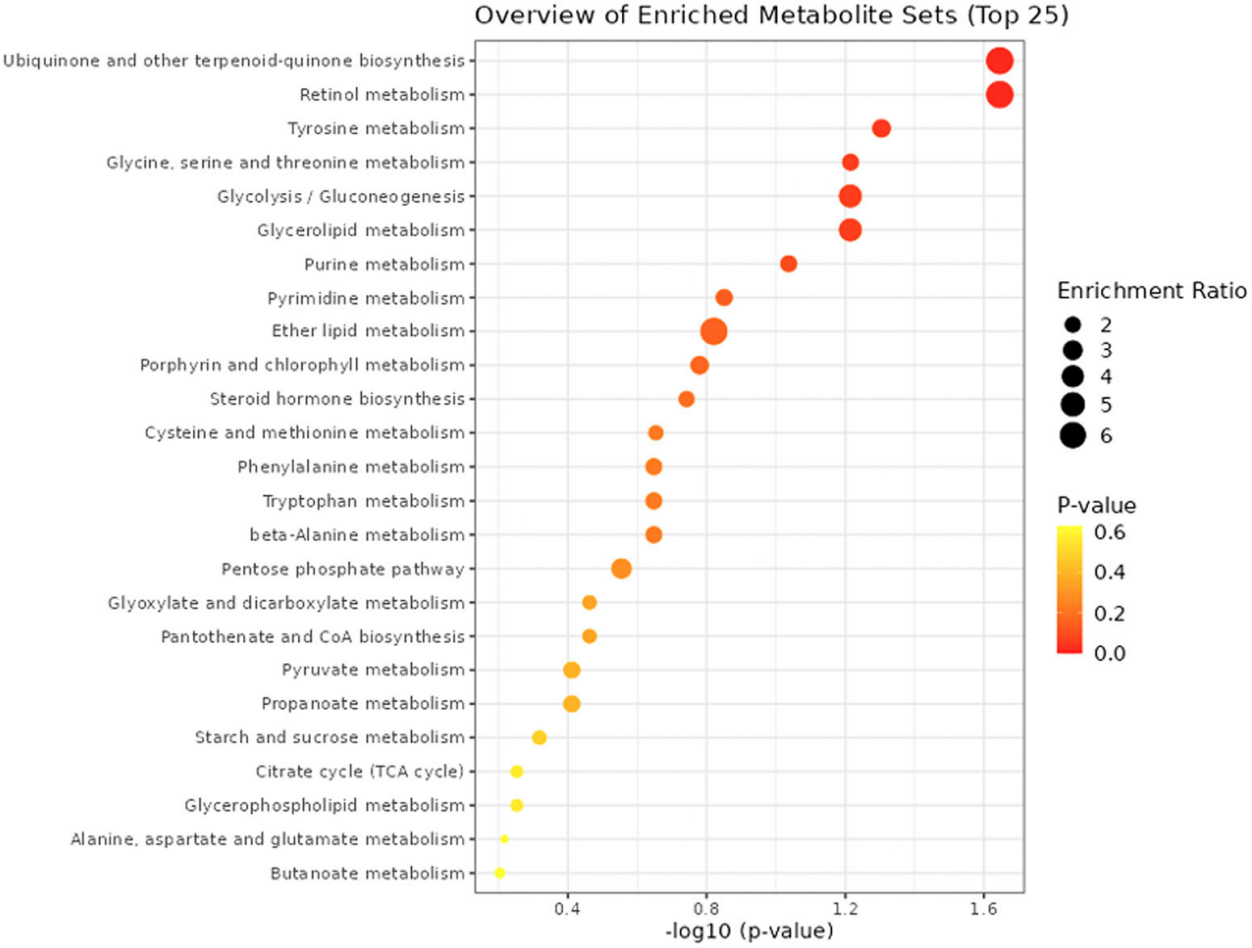
Dot plot visualization of pathway enrichment analysis results. Dot plot visualization of pathway enrichment analysis results for the de novo PD vs. control comparison using the KEGG database. The horizontal axis shows the negative decadic logarithm of the *p* value, and the vertical axis shows the pathways, sorted by decreasing significance from the top. The color gradient from red to yellow reflects increasing *p* values, and the size of the dots reflects the effect size for each pathway (enrichment ratio; see the legend on the right).

For both databases only nominally significant pathways were identified, including “Fatty Acids and Conjugates” (*p* value = 1.9E-3, adj. *p* value = 0.25), and “Fatty Acyls” (*p* value = 4.96E-02, adj. *p* value = 1) for the chemical structure classes (see Fig. 2 and Supplementary Table 9), and “Ubiquinone and other terpenoid-quinone biosynthesis” (*p* value = 2.26E-02, adj. *p* value = 1), “Retinol metabolism” (*p* value = 2.26E-02, adj. *p* value = 1), and “Tyrosine metabolism” (*p* value = 4.96E-02, adj. *p* value = 1) for the KEGG database (see Fig. 3 and Supplementary Table 10).

Changes in “Fatty Acids and Conjugates” and “Fatty Acyls” match with the statistically significant alterations in individual metabolites in fatty acid metabolism already discussed (see section “Fatty acid metabolism / β-oxidation”). A suppression of mitochondrial fatty acid ß-oxidation in an early stage of PD (Hoehn and Yahr stage I), characterized by decreased levels of long-chain acylcarnitine, has been described before and proposed as a potential diagnostic biomarker for PD^34^. While the mechanism behind this dysregulation is still unclear, an overexpression of a mutant form of the gene encoding alpha-synuclein (A53T-SNCA) linked to familial cases of PD has been shown to increase triacylglycerol levels and associated with increased activity of acyl-CoA synthetase, which catalyzes fatty acyl-CoA formation as a substrate for β-oxidation^47^.

The identified nominally significant alteration in “Retinol metabolism” matches with the observed change for the carotenoid retinal (see section “Catecholamine metabolism (retinal, ALDH1A1)”), while the changes for “Ubiquinone and other terpenoid-quinone biosynthesis” and “Tyrosine metabolism” may both reflect alterations in the network around the amino acid L-tyrosine, which is involved in both of these KEGG pathways and displays a nominally significant change in de novo PD vs. controls (*p* = 0.013).

Overall, the pathway enrichment analysis results, while only providing nominally significant findings, match with the relevance of the functional annotations for individually significant metabolites involved in fatty acid β-oxidation and indicate further putative changes in the network around L-tyrosine for follow-up study.

### Comparison with previously reported metabolomic pathway alterations in PD

To assess the consistency of our findings with previously reported metabolomics cellular process alterations in PD, we have compared the main coordinated changes in the PD blood metabolomics biomarker identification study by Hatano et al.^55^ with our results. The experimental set-up used by Hatano et al. differed from our approach in multiple relevant aspects: Serum samples were analyzed instead of plasma samples, the study focused on patients who had already received antiparkinsonian treatment (35 subjects) and age-matched healthy controls (15 subjects), and it used measurements from ultrahigh-performance liquid chromatography/tandem mass spectrometry (UPLC/MS/MS) optimized for basic species, UPLC/MS/MS optimized for acidic species, and gas chromatography/MS (GC/MS). Despite these methodological differences, when comparing the main pathway alterations in this prior study with our liquid chromatography–mass spectrometry (LC-MS) metabolomics data for all PD patients vs. controls, we observe largely consistent qualitative results.

**Fig. 4:**
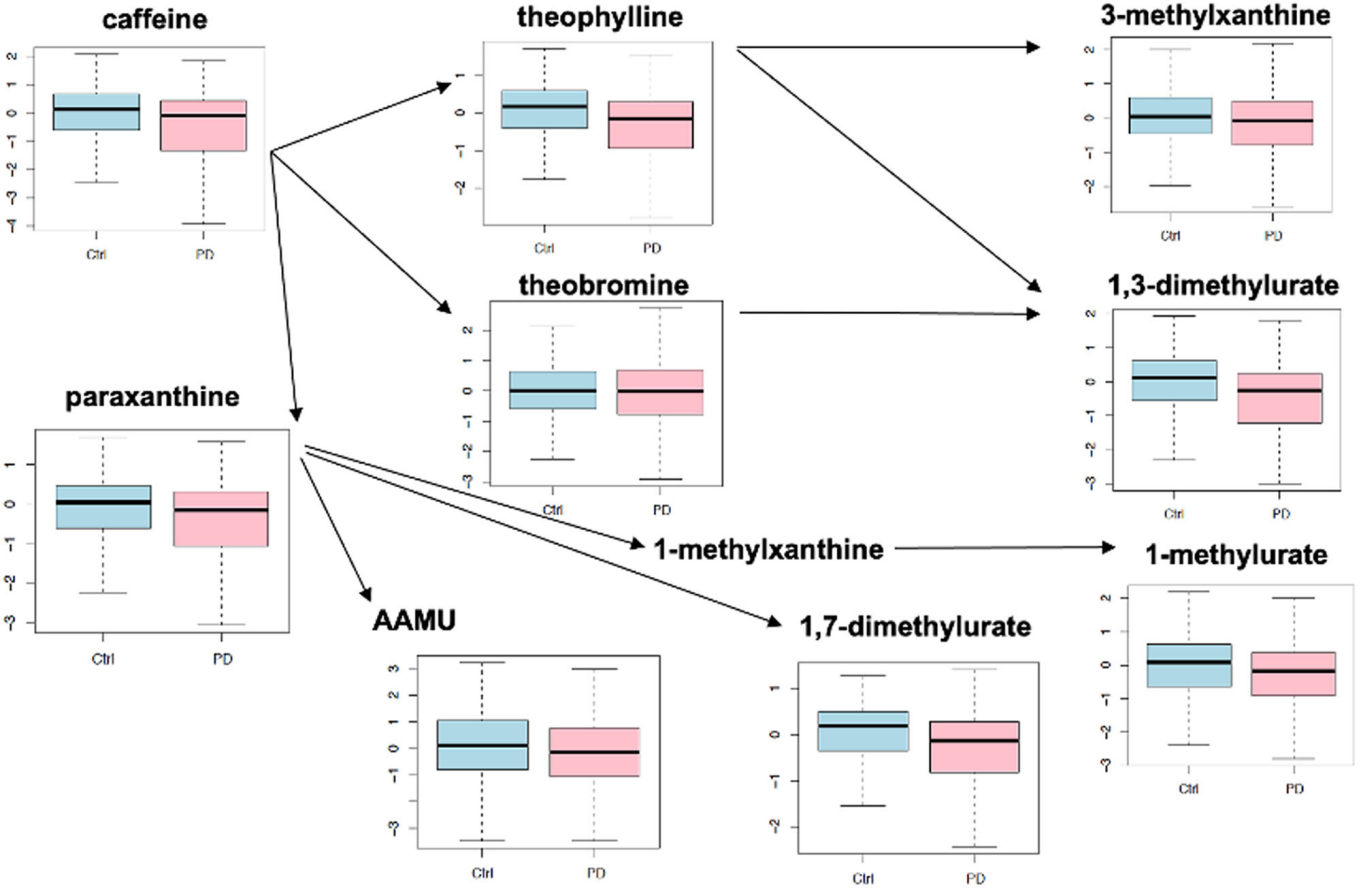
Box plot visualization of alterations in caffeine metabolism. Box plots showing alterations in caffeine metabolism in PD vs. controls, reproducing the results reported in the study by Hatano et al. (see Fig. 2 in Ref. ^55^, which was used as a model). Vertical axes represent log-scale normalized abundances, and the horizontal axes show the two conditions: control (Ctrl, in blue) and PD (in pink). Arrows indicate enzymatic reactions which relate the source metabolites to their conversion products (the source of the arrows represent the educts, and the arrow targets the products).

In particular, as a key finding, Hatano et al. report that the levels of caffeine and its main metabolites were consistently lower in PD than in controls, which matches our observations (see Fig. 4, which seeks to reproduce the visualization of changes in caffeine metabolism presented in Fig. 2 in the study by Hatano et al.^55^).

Although the corresponding effect sizes are small and only nominal significance is observed in most cases, our metabolomics data confirms a consistent pattern of average decreased caffeine metabolite abundances in PD. However, the nominally significant decrease in caffeine levels in treated patients vs. controls (*p* = 0.033) is not observed in the de novo patients vs. controls (*p* = 0.7). These findings match with prior knowledge on complex associations between caffeine intake and PD. On the one hand, caffeine has been reported to have neuroprotective effects and has been associated with a lower risk of developing PD^14^. On the other hand, as a central nervous system stimulant, it could potentially worsen PD symptoms, such as tremors, possibly resulting in reduced caffeine intake among patients. Furthermore, interactions between dopaminergic medication and caffeine may occur^56^, which could explain why the nominally significant decrease in caffeine levels in all PD patients vs. controls is not seen in de novo PD vs. controls. Further research will be essential to understand these variation patterns, considering factors such as dietary caffeine intake, symptom profiles, and specific dopaminergic treatments.

Interestingly, the same pattern of nominally significant alterations specific to treated patients is also observed for caffeine metabolites, including multiple xanthine alkaloids such as paraxanthine (treated PD vs. controls: *p* = 0.015; de novo PD vs. controls: *p* = 0.47), 1-methylxanthine (treated PD vs. controls: *p* = 0.02; de novo *PD* vs. controls: *p* = 0.78), and 7-methylxanthine (treated PD vs. controls: *p* = 0.029; de novo *PD* vs. controls: *p* = 0.33, see Supplementary Tables 5 and 7). Thus, while the xanthines involved in purine metabolism (inosine, xanthosine, xanthine, and hypoxanthine) display a significantly increased abundance in de novo PD vs. controls (see Table 3 and discussion above), xanthine alkaloids involved in caffeine metabolism only display nominally significant changes in treated patients, with a trend of decreased abundances consistent with their predecessor metabolite caffeine. This matches with prior data indicating that interactions between caffeine metabolism and dopaminergic treatment effects need to be considered, as caffeine intake has been reported to shorten the maximal plasma concentration of L-DOPA^56^.

**Fig. 5:**
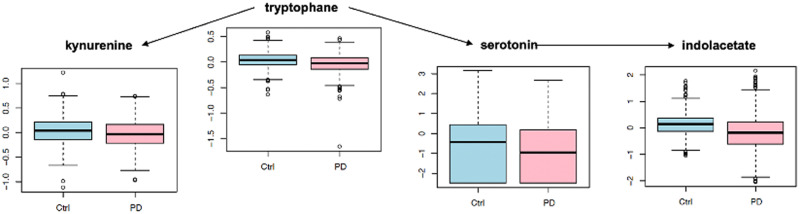
Box plot visualization of alterations in tryptophan metabolism. Box plots showing alterations in tryptophan metabolism in PD vs. controls, reproducing the results reported in the study by Hatano et al. (see Fig. 1B in Ref. ^55^, which was used as a model). Vertical axes represent log-scale normalized abundances, and the horizontal axes show the two conditions: control (Ctrl, in blue) and PD (in pink). Arrows indicate enzymatic reactions which relate the source metabolites to their conversion products (the source of the arrows represents the educts, and the arrow targets the products).

As a second main pathway alteration, Hatano et al. reported changes in tryptophan metabolism, with a pronounced reduction in tryptophan levels and slight reductions in some of its downstream conversion products. Our study observed a similar trend in directional change, but the effect sizes for individual metabolites were not large enough to reach statistical significance (see Fig. 5, which qualitatively reproduces the directional changes from Fig. 1B in the study by Hatano et al.).

In line with previous reports of changed tryptophan levels in L-DOPA treated rats^57^, alterations in tryptophan metabolism may mainly represent an L-DOPA treatment effect and not a disease-specific change. However, we note that tryptophan displayed at least a nominally significant decrease (*p* = 0.003) for the comparison of de novo PD vs. controls. Independent of the cause, the observation of decreased tryptophan levels in PD may be relevant for the choice of adjuvant treatments, as reduced plasma and serum levels of tryptophan have been linked with depression by multiple studies^58,59^.

Overall, we observe qualitatively similar results for treated patients in this study compared to the main pathway alterations reported by Hatano et al., indicating coordinated changes in caffeine and tryptophan metabolism. Additional studies will be required to assess the physiological implications of these changes and their precise relationship with PD medication.

### Metabolite associations with MDS-UPDRS motor scores

To examine if blood metabolite levels are linked to the severity of movement impairments in Parkinson’s Disease (PD), as measured by the MDS-UPDRS-III (Movement Disorder Society-Unified Parkinson’s Disease Rating Scale Part III), we fitted linear models, adjusting for sex, age, and L-DOPA medication effects.

The results showed that eight metabolites were significantly associated with the MDS-UPDRS-III after adjusting *p* values for multiple hypothesis testing (see Table 4). Except for one unidentified metabolite, all displayed negative correlations with MDS-UPDRS-III scores with small effect sizes. Five of the seven significant metabolites with known identity are caffeine-related metabolites, including caffeine itself and its conversion products theophylline, paraxanthine, 1,3,7-trimethylurate, and 1,7-dimethylurate. Considering this result together with our observation of consistently lower levels of caffeine metabolites in PD vs. controls discussed above and the qualitatively similar results reported by Hatano et al.^55^, the inverse association of these metabolites with both the presence of PD and motor severity matches with their protective role as suggested in epidemiological studies and experimental models (see section on “Xanthine metabolites”). However, it is important to also consider the possibility of inverse causation, e.g., severe motor symptoms might lead to behavioral changes including reduced caffeine consumption. Additionally, a potential influence of other PD-related molecular factors on caffeine metabolites cannot be excluded. Interestingly, a previous study that monitored both total caffeine intake and measured serum levels of caffeine and nine of its downstream metabolites in 108 PD patients and 31 age-matched healthy controls concluded that the observed significantly decreased levels of these metabolites were unrelated to total caffeine intake and may serve as a potential diagnostic biomarker signature^60^.

**Table 4:** Ranking of metabolites with significant associations with MDS-UPDRS-III scores in PD patients

Metabolite	Spearman’s rho	*P* value	Adj. *P* value
2 S,3R-dihydroxybutyrate	−0.13	3.30E-07	4.91E-04
Theophylline	−0.18	2.52E-06	1.88E-03
Paraxanthine	−0.15	4.78E-06	2.00E-03
Caffeine	−0.16	5.38E-06	2.00E-03
X-23648	**0.22**	2.35E-05	7.00E-03
1,3,7-trimethylurate	−0.13	4.71E-05	1.17E-02
Behenoyl sphingomyelin	−0.23	6.38E-05	1.36E-02
1,7-dimethylurate	−0.16	9.53E-05	1.77E-02

The columns show the metabolite names (Metabolite), the Spearman correlation between metabolite abundance levels and the MDS-UPDRS-III (Spearman’s rho), the nominal *p* value significance (*P* value) and the adjusted *p* value (adj. *P* value). In addition to the column headers highlighted in bold, for metabolites with positive Spearman correlations, the value of Spearman’s rho is shown in bold, and for metabolites with negative correlations, in regular font.

Besides caffeine metabolites, only two other metabolites, 2 S,3R-dihydroxybutyrate and behenoyl sphingomyelin, also showed significant negative associations with the MDS-UPDRS-III score. 2 S,3R-dihydroxybutyrate (also known as 4-deoxythreonic acid) is a secondary metabolite and sugar acid. It has been described as an L-threonine metabolite which correlates negatively with age in adults^61^, but has to our knowledge not been linked directly to PD. The last metabolite with a negative association, behenoyl sphingomyelin, is a sphingolipid contained in animal cell membranes, in particular in the myelin sheath surrounding certain nerve cell axons^25^. Sphingomyelins have been implicated in several cellular processes with potential relevance in PD, including nerve impulse transmission, presynaptic plasticity, and the localization of neurotransmitter receptors^62^. Furthermore, intralysosomal accumulation of sphingomyelins is a pathological mechanism in lysosomal storage disorders, such as Gaucher disease and Niemann-Pick disease, which are both associated with an increased risk of developing PD^62^. A proposed mechanism linking these disorders to PD is that sphingolipid changes promote a pathological conversion of alpha-synuclein into a proteinase K-resistant conformation and induce its oligomerization^63^. However, while saturated sphingomyelin species are depleted in the putamen of *post-mortem* tissue samples from PD patients^64^, the processes involved remain under investigation.

Overall, MDS-UPDRS-III associations were identified for eight metabolites, which are predominantly involved in caffeine metabolism (no further pathway enrichment analysis was therefore conducted in this case). The results match with the findings on altered caffeine metabolism in the PD vs. control comparison.

### Integrative network analysis of metabolomic and transcriptomic changes in PD

**Fig. 6:**
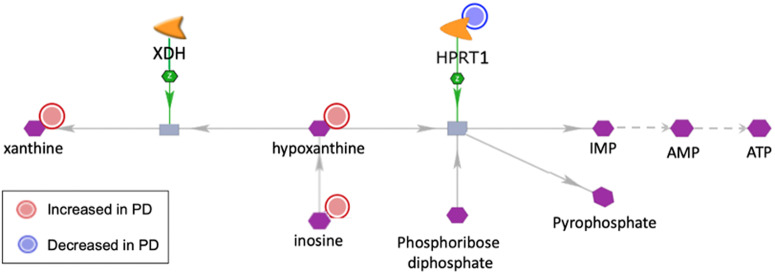
Molecular sub-network visualization of alterations in xanthine metabolism. Molecular sub-network visualization, highlighting shared alterations in xanthine metabolism identified by a joint network analysis of PD vs. controls transcriptomics and metabolomics data (increased abundances are highlighted by red circles, decreased abundances by blue circles). The network analysis suggests decreased expression of the gene *HPRT1* as the main cause for an accumulation of the metabolite hypoxanthine and further related xanthines in PD.

To better understand the molecular mechanisms behind the observed metabolite alterations, we performed an integrated molecular network analysis of the metabolomics data with complementary PD case/control transcriptomics data. In line with the statistical findings for individual metabolites, this analysis identified a coordinated sub-network alteration associated with xanthine metabolism. Specifically, it highlighted a regulatory consistent sub-network change with increased abundances in three xanthine metabolites (xanthine, hypoxanthine, and inosine) and decreased gene expression for the associated enzyme hypoxanthine phosphoribosyltransferase 1 (*HPRT1*), which catalyzes the conversion of hypoxanthine and phosphoribose diphosphate into inosine monophosphate (IMP) and pyrophosphate (see Fig. 6).

Given the role of *HPRT1* in hypoxanthine conversion, the decreased *HPRT1* expression in PD may contribute to the increased abundances of xanthines by inhibiting the processing of hypoxanthine and its precursor inosine through this branch of the conversion pathway (see Fig. 6, right side), and thereby indirectly increase the levels of the alternative hypoxanthine conversion product xanthine (see Fig. 6, left side). Importantly, the relevant xanthines (inosine, hypoxanthine and xanthine) all pass the blood-brain-barrier^65–67^, and in patients with an *HPRT1* deficiency, both plasma and CSF levels of xanthine and hypoxanthine are elevated compared to controls^68,69^, indicating that changes in plasma xanthine abundances may indeed be linked to *HPRT1* expression changes in the brain. Interestingly, the most severe human form of *HPRT1* deficiency, known as Lesch-Nyhan’s syndrome and caused by *HPRT1* mutations, is a neurological disorder characterized by hyperkinetic movements and by loss of dopamine in the basal ganglia. This suggests that the PD-associated changes we identified in *HPRT1* and in the associated xanthines may reflect a mechanism relevant to basal ganglia dysfunction in PD.

While xanthines have been linked to multiple molecular mechanisms in PD, including the generation of reactive oxygen species by xanthine oxidase and the inhibition of the adenosine A2A receptor and Monoamine Oxidase type B (see section on “Xanthine metabolites”), the observed sub-network alteration involving decreased *HPRT1* expression suggests an additional mechanism by which xanthines may influence PD. Specifically, the hypoxanthine conversion product inosine monophosphate (IMP), resulting from the reaction catalyzed by *HPRT1*, is a precursor for adenosine monophosphate (AMP), which in turn is required for the synthesis of adenosine triphosphate (ATP, see Fig. 6, right side). Thus, diminished HPRT1 expression in Parkinson’s Disease (PD) and the resultant lower conversion of hypoxanthine to inosine monophosphate (IMP) may contribute to a deficiency of cellular ATP in PD. Indeed, rare genetic mutations in the HPRT1 gene, linked to Lesch-Nyhan syndrome, are known to lead to elevated xanthine levels and a concurrent reduction in cellular ATP^70^. Defects in cellular ATP production found in several regions of PD brains have been associated with mitochondrial dysfunction^71^, and previous studies showing that *HPRT1* deficiency inhibits mitochondrial protein complex I-dependent respiration^72^ indicate that *HPRT1* may be involved in energy metabolism dysregulations in PD. Additionally, a further independent study reports that *HPRT1* exhibits decreased expression in PD and in other neurodegenerative disorders involving mitochondrial dysfunction, including Alzheimer’s and Huntington’s disease^73^.

Interestingly, in an open-label, single-arm trial, 26 PD patients received a combined treatment with inosine and an inhibitor of the enzyme XDH that converts hypoxanthine to xanthine (see Fig. 6, left side), and this treatment increased blood hypoxanthine and ATP levels, and lowered the patients’ UPDRS III motor impairment score^74^. Considering the mechanisms linking hypoxanthine to ATP metabolism as outlined in Fig. 6, *HPRT1* may therefore warrant further study as a potential alternative target for pharmacological induction to achieve similar treatment effects as the combination of inosine and XDH inhibition.

Finally, *HPRT1* has been linked to PD also through its role in activating Wnt/ß-catenin signaling, a pathway known to protect dopaminergic neurons in the 6-hydroxydopamine mouse model^75^. Reduced *HPRT1* gene and protein expression was observed in the *substantia nigra* of these mice, in line with our observations in human PD, and lentiviral over-expression of *HPRT1* in this model inhibited neuron loss. Interestingly, the study also suggested a possible upstream mechanism for *HPRT1* under-expression in PD by showing that LncRNA H19, which is also under-expressed in PD, normally elevates *HPRT1* expression by inhibiting miR-301b-3p. Over-expressing H19 not only raised *HPRT1* levels but also activated Wnt/β-catenin signaling, reducing neuron loss. Thus, multiple lines of evidence suggest protective effects of rescuing *HPRT1* under-expression in PD.

In summary, the observed under-expression of *HPRT1* in PD and associated increases in the levels of xanthines may influence disease pathways via multiple independent mechanisms. Follow-up studies in independent human biospecimens and complementary disease models will need to further confirm and characterize the clinical relevance of these mechanisms and associated rescue strategies.

### Machine learning analysis

Classification and regression analyses were conducted to identify potential metabolite markers helping to distinguish de novo PD and control subjects (see Table 5) and to predict motor symptom severity in treated patients, as indicated by the MDS-UPDRS III total score (see Table 6).

**Table 5:** Ranking table of the top 10 known metabolite features using supervised sample classification

Metabolite	Linear AUC (Train)	Linear AUC (Test)	Radial AUC (Train)	Radial AUC (Test)	Average AUC
Xanthine	0.68	0.71	0.63	0.72	0.68
2-ketocaprylate	0.58	0.74	0.54	0.78	0.66
Glutarylcarnitine (C5-DC)	0.64	0.68	0.62	0.66	0.65
4-hydroxyphenylpyruvate	0.57	0.68	0.61	0.66	0.63
N-palmitoyl-sphingadienine (d18:2/16:0)	0.57	0.58	0.70	0.65	0.63
3-hydroxyoleoylcarnitine	0.57	0.62	0.64	0.68	0.62
3-hydroxybutyroylglycine	0.61	0.62	0.66	0.60	0.62
Cortisol	0.53	0.70	0.56	0.69	0.62
Hypotaurine	0.60	0.66	0.59	0.63	0.62
Palmitoleoylcarnitine (C16:1)	0.59	0.65	0.57	0.66	0.62

Ranking table of the top 10 known metabolite features in terms of their estimated predictive value for supervised sample classification of de novo PD and control samples, showing each metabolite feature (column Metabolite), the Area Under the ROC Curve (AUC) for the training set (columns Train_Linear_AUC, Train_Radial_AUC) and for the test set (columns Test_Linear_AUC, Test_Radial_AUC) for both linear and radial Support Vector Machine (SVM) classifiers. Rows are sorted by the average AUC across training and test set AUC scores for both linear and radial SVMs (column Average_AUC, all column headers are highlighted in bold). The complete ranking table, including the ranking scores for unidentified metabolites, is provided in Supplementary Table 10.

The top three identified metabolites for distinguishing de novo PD from controls were xanthine, 2-ketocaprylate, and glutarylcarnitine, as indicated by their higher average area under the Receiver Operating Characteristic Curve (AUC) values across both linear and radial support vector machine (SVM) classifiers in training sets (5-fold cross-validation) and test sets (see Table 5; a complete ranking table, including the AUC values for unidentified metabolites, is provided in Supplementary Table 11). Xanthine, in particular, shows consistent performance in both classifiers, with its test set AUC scores (0.71 for the linear SVM, 0.72 for the radial SVM) slightly exceeding the cross-validated training scores (0.68 for the linear SVM, 0.63 for the radial SVM). This result is consistent with the changes in xanthine metabolism observed in the statistical and network analyses, although we note that it should not be considered as validation, as the same input data was used for these analyses. By contrast, 2-ketocaprylate and glutarylcarnitine were not statistically significant in the de novo PD vs. controls differential analysis, showing that the machine learning-based feature ranking offers distinct information from the statistical analyses. Predictive changes in these two metabolites may reflect disturbances in branched-chain amino acid metabolism and mitochondrial function, respectively, as 2-ketocaprylate is a branched-chain alpha-keto acid that serves as an intermediate of branched-chain amino acids^25^, and glutarylcarnitine is an acylcarnitine and an intermediate in lysine and tryptophan metabolism^76^, processes that rely heavily on mitochondrial efficiency.

In the regression analysis results (see Table 6, dopamine metabolites, such as homovanillate (HVA) and various dopamine sulfates, rank highest among the metabolite features for predicting MDS-UPDRS III total motor scores, indicated by the lowest sum-of-ranks across the performance metrics for both linear and radial SVM regression models (a complete ranking table, including scores for unidentified metabolites, is provided in Supplementary Table 12). The presence and levels of these dopamine-related metabolites are likely influenced by dopaminergic medication and may reflect increases in medication intake for patients impacted by more severe motor impairments. While an accurate prediction model for UPDRS III total motor scores would still have practical value, the coefficient of determination (R²) is generally low even for the top-ranked features, suggesting a limited predictivity. However, the occurrence of sphingomyelin among the top-ranked features matches with the significant UPDRS III total score association observed for behenoyl sphingomyelin in the statistical analyses and with previous studies on functional implications of sphingomyelins in PD (see section on “Metabolite associations with MDS-UPDRS motor scores”). Thus, potential links between sphingomyelin metabolism and PD motor impairment may warrant further study.

Overall, the classification and regression analyses of highlighted metabolites as most predictive that match with prior associations identified for xanthines and sphingomyelins in the statistical analyses. However, metabolites only provided limited predictive information when used individually, and further analyses are needed to assess whether a panel of multiple metabolites used in combination could potentially enhance predictive power. Follow-up machine learning analyses for multivariable predictive modeling, integration of metabolomics data with complementary data types (clinical, omics, digital biomarkers, polygenic risk scores, among others), and prediction of further disease outcomes (motor and non-motor scores, comorbidities), which would extend beyond the scope of the current manuscript, are currently in preparation.

### Study limitations

This study recognizes limitations inherent in its design and methodology.

### Assessment of diagnostic status

As an observational study, the Luxembourg Parkinson’s Study relies on imaging performed during routine diagnostic examinations by patients’ treating physicians. The inclusion of DaTscan (Dopamine Transporter Scan) and other structural imaging in our dataset supports the diagnostic assessments made by the study physicians. In addition, the annual longitudinal follow-up of patients enhances the diagnostic confidence by monitoring for sustained dopaminergic response and the absence of warning signs that might lead to reconsideration of the diagnosis and possible reclassification to atypical or secondary parkinsonism. Despite these measures, a small proportion of misclassifications, particularly in the early stages of PD and in de novo patients, cannot be completely excluded. Such misclassifications are not expected to significantly affect the qualitative results of our study, which consistently includes more than 50 subjects per group.

### Noise, bias, and confounding

Regarding possible sources of noise, bias, and confounding in the data, we note that we did not record the time between the last meal and blood sampling, and we did not sample after overnight fasting. Blood levels of glucose, lipids, and amino acids, among other metabolites, can vary significantly with dietary intake, and in this study, we did not have the means to ensure that patients and controls received the same diet or participated in the fasting state. Therefore, potential systematic differences in diet between the study groups could also lead to differences in the metabolite profiles. For this reason, we have discussed potential dietary influences and gut microbiome influences in our interpretation of the individual metabolite changes (see section on ”Significant metabolite changes in de novo Parkinson’s disease and treated patients”). In general, these factors may contribute to greater variability in the data, underscoring the need for a cautious interpretation of the results.

It is important to note the time lag between blood collection and processing: Samples collected in the morning (between 9 am and 12 pm) were delivered to the biobank by ~1 pm and processed within 1–1.5 hours, and derivatives were immediately frozen at −80 °C (pre-centrifugation delay between 1 and 4 h); samples collected in the afternoon (between 12 pm and 5 pm) were delivered to the biobank by ~9 am the next day and processed within 1–1.5 hours, and derivatives were immediately frozen at −80 °C (pre-centrifugation delay between 16 and 21 h). There were no systematic differences in the handling of samples from different study groups, and accordingly, no systematic differences in postprandial status or circadian cycle are expected between the groups. However, stochastic variation in these parameters and the time lags between sample collection and further processing are limitations that may increase measurement variance. All samples underwent two freeze-thaw cycles prior to analysis, and any potential bias associated with this should be the same for all samples. To identify blood samples and metabolomics measurements of insufficient quality for further processing and analysis, multiple quality control analyses were performed on both blood and metabolomics samples, including quality control samples such as a solvent blank and ultrapure water as a process blank, and multiple quality control standards (described and justified in detail in the Methods sections “Biospecimen collection, quality control and sample accessioning” and “Metabolomics sample processing”).

Finally, we note that as the samples were randomly assigned across the measurement batches in combination with samples from another independent study, balanced sample group representations for the present study could not always be ensured across the batches, which may have led to increased data variance.

### Missing value imputation and normalization

Missing values were imputed using the minimum detected value, following Metabolon’s routine approach. Since missing values in this type of data are generally the result of data falling below the limit of detection, the data in these cases are left-censored with informative missingness. In total, 21.9% of the values were missing and imputed, but as they reflect data falling below the detection limit, the percentage of missing values is not an indicator of data quality in this setting. There were no significant differences in the percentage of imputed values between the study groups (before imputation, controls had 21.8% missing values, PD samples had 22.0% missing values, and de novo PD samples had 21.4% missing values). Because the imputation filled in missing values using the minimum observed values, and because the proportion of missingness was very similar across the batches, there was no significant change in the individual median values and in the differences between the medians, and no renormalization was required. The possible alternative approach of normalizing with quality control samples was not chosen, because it relies on fewer samples, which could potentially reduce the robustness of the normalization.

### Adjustment for treatment effects

For the study of PD patients receiving dopaminergic treatments, it is important to note that using 3-O-Methyldopa (3-OMD, 3-methoxytyrosine) as a covariate to adjust for treatment effects has limitations. 3-OMD is an imperfect indicator of the many different types of dopaminergic medications taken by PD patients and can be modulated by both catechol-O-methyltransferase (COMT) inhibitors and the prolonged drug effects of carbidopa. L-DOPA itself was not a covered metabolite in this LC-MS profiling study and therefore could not be used instead of 3-OMD. Therefore, adjustment for 3-OMD in the data for treated patients is not expected to fully correct for the confounding effects of dopaminergic medications, and the separate analysis of de novo patients is an essential component of this profiling study.

### Group differences in cognitive impairment

Significant group differences in cognitive decline, as assessed by the MoCA (Montreal Cognitive Assessment) score, were observed in PD patients compared with controls (see Table 3). Although there is evidence that PD can cause cognitive decline, e.g., through a dysfunction of fronto-striatal pathways^77^, to our knowledge there is no clear evidence suggesting that early cognitive decline increases the risk of developing PD. A variable that is affected by the outcome does not meet the definition of a confounder, and by adjusting for a covariate that predominantly represents a downstream effect of the disease, we would risk removing the primary effects of the disease itself. However, we note that some of the observed metabolite changes may not be directly associated with PD, but rather reflect cognition-related changes. Nevertheless, differences in MoCA scores between cases and controls cannot affect our association analysis of motor scores (MDS-UPDRS III), which is focused exclusively on PD patients.

### Machine learning analyses

Given the limited sample size available for de novo PD patients (56 subjects), the impact of dopaminergic medications on blood metabolite levels in treated patients, and the focus on a single cohort, the machine learning and cross-validation results herein should be considered as preliminary estimates of the achievable predictive power for case-control classification and MDS-UPDRS III total score prediction. Furthermore, we focused on single metabolites as predictors and the potential of multi-variable signatures to improve the predictive performance will require further investigation. In general, for both the optimization and robust validation of metabolite-derived machine learning models, independent analyses with larger sample sizes of de novo patients across multiple distinct cohorts are needed.

## Discussion

The study of blood plasma metabolomics for the Luxembourg Parkinson’s Study revealed several statistically significant disease-associated changes. In statistical and machine learning analyses of de novo PD patients compared to controls, xanthine metabolites stood out among the most significant and predictive metabolites. The xanthine alteration patterns showed consistency and coordination both in terms of the direction of changes, with a common increased abundance for inosine, xanthosine, xanthine, and hypoxanthine in de novo PD vs. controls, and in terms of the mechanistic matching of changes with complementary PD vs. control transcriptomics data in an enzyme-metabolite regulatory network analysis. As a main finding, the integrated network analysis also highlighted a potential key enzyme, *HPRT1*, which catalyzes the conversion of hypoxanthine to IMP, and whose decreased abundance in PD may explain the increased metabolite abundances observed in the alternative hypoxanthine-to-xanthine pathway (see Fig. 6).

While the case-control statistical comparison of metabolomics data cannot distinguish causal from non-causal relationships, the coordinated regulatory network changes identified in xanthine metabolism point to potential clinically relevant disease mechanisms, paving the way for targeted follow-up validation studies. Furthermore, the detected coordinated sub-network changes may have relevant applications in biomarker signature modeling for PD due to their consistency and robustness across multiple biomolecules in two different omics data types. In this context, we note that in a previous study of CSF biomarkers in an independent PD cohort, the metabolites xanthine, homovanillic acid, and their ratio were proposed as biomarkers of PD status and severity^78^, suggesting that xanthine is altered in multiple body fluids in PD and may indeed have diagnostic applications.

In addition to biomarker applications, xanthines and their derivatives, including naturally occurring xanthine alkaloids such as caffeine, paraxanthine, and theophylline, have previously been proposed as potential neuroprotective agents for PD. Multiple studies using in vitro and in vivo models of PD have confirmed protective effects of these compounds^14,16,79^, e.g., for paraxanthine, caffeine, and theophylline in an MPTP mouse model^14^, and for inosine in a cellular PD model^80^. While the main proposed protective mechanism for inosine and other xanthines links them to the downstream metabolite uric acid as a mediator of neuroprotective effects via induction of the Nrf2 signaling pathway^81^, other studies suggest that the protection by inosine is independent of uric acid^80^. Further alternative candidate protective mechanisms for xanthines and their derivatives include inhibition of the adenosine A2A receptor and the enzyme MAO-B, both of which are well-established PD drug targets with associated clinically approved drugs (e.g., the xanthine derivative drug Istradefylline, which targets A2A^82^, and the MAO inhibitor selegiline^83^).

In addition to these known actions of xanthine derivatives, the integrated metabolomic and transcriptomic network analysis performed here suggests another possible pathway by which xanthine alterations may affect PD-related processes. The observed decreased expression of the enzyme *HPRT1*, which converts hypoxanthine to IMP, provides both a mechanistic explanation for the observed increased abundance of xanthines in PD and is associated with a shortage of cellular ATP as a further pathological downstream effect (see Fig. 6). This potential disease mechanism is supported by a previous single-arm, open-label trial in PD patients, targeting the same pathway by a combined treatment with inosine and an inhibitor of the enzyme XDH to block the conversion of hypoxanthine to xanthine^75^. This inhibition favors the conversion of hypoxanthine to IMP by HPRT1 and has been shown in the same study to increase downstream ATP production and improve PD motor symptoms.

Besides the main finding of coordinated changes in xanthines, the metabolite analyses also revealed significant alterations in other PD-related cellular processes. In particular, pronounced changes in fatty acid β-oxidation were observed, consistent with previous studies reporting impaired mitochondrial β-oxidation in PD^34^. In addition, the combined analysis of metabolomics and transcriptomics data revealed coordinated changes in catecholamine metabolism, with significantly increased levels of the metabolite retinal coinciding with a decreased expression of the enzyme aldehyde dehydrogenase 1A1 (*ALDH1A1*), which converts retinal to retinoic acid. These findings are in line with the catecholaldehyde hypothesis of PD, which links reduced ALDH1A1 levels to toxic accumulation of the dopamine metabolite DOPAL^19,20^.

The machine learning analyses for de novo PD vs. control classification and for prediction of MDS-UPDRS III total motor score provided results that matched with the associations for xanthines and sphingomyelins observed in the statistical analyses. However, the predictive capacity of individual metabolites was low to moderate, and further analysis is needed to evaluate potential improvements of multi-metabolite signatures and confirm the results across different cohorts.

Overall, the investigations revealed several significant metabolite changes in de novo PD and treated patients. These findings highlight coordinated and mechanistically congruent alterations in specific cellular processes and sub-networks, laying the ground for follow-up mechanistic intervention and validation studies in PD model systems.

## Methods

### Study cohort

The data used in this study were obtained from participants recruited from the nationwide, monocentric, observational, longitudinal Luxembourg Parkinson’s Study^10^ in the frame of the National Centre of Excellence in Research on Parkinson’s disease (NCER-PD). All subjects signed a written informed consent, and the study was approved by the National Research Ethics Committee (CNER Ref: 201407/13) and complied with all relevant ethical regulations. To characterize the cross-sectional metabolite alterations in PD, blood plasma samples were obtained from 549 PD patients and 590 controls from the Luxembourg Parkinson’s Study and submitted for metabolomics profiling (study approved by the University of Luxembourg Ethics Review Panel, ref. ERP 18-042). An overview of relevant baseline subject characteristics for PD patients and controls from this dataset is shown in Table 3. The clinical diagnosis of PD adhered to the United Kingdom Parkinson’s Disease Society Brain Bank (UKPDSBB) diagnostic criteria^84^. Criteria for the inclusion of controls in the Luxembourg Parkinson study were: (i) no evidence of neurodegenerative disorders according to all clinical assessments and imaging information available to the assessing study physician; (ii) age above 18 years, (iii) absence of pregnancy or active cancer. De novo PD was defined as dopaminergic treatment-naïve patients within one year of PD diagnosis.

**Table 6:** Ranking table of the top 10 known metabolite features for regression analysis of motor scores

Metabolite	Linear RMSE	Linear R²	Radial RMSE	Radial R²	Sum of ranks
Homovanillate (HVA)	13.74	0.07	13.76	0.07	11
Dopamine 3-O-sulfate	13.80	0.07	13.80	0.07	16
Vanillactate	13.74	0.06	13.73	0.06	17
3-methoxytyramine sulfate	13.81	0.06	13.81	0.06	30
Dopamine 4-sulfate	13.83	0.05	13.82	0.06	36
Sphingomyelin	13.90	0.05	13.82	0.06	45
Urea	13.88	0.04	13.99	0.05	64
3-methoxytyrosine	13.74	0.07	14.10	0.06	70
4-acetamidobenzoate	13.88	0.05	14.07	0.05	78
11-ketoetiocholanolone glucuronide	13.97	0.04	14.02	0.04	95

Ranking table of the top 10 known metabolite features in terms of their estimated predictive value for regression analysis of UPDRS III total motor scores in treated PD patients, displaying each metabolite feature (column Metabolite), the Root Mean Square Error (columns “Linear RMSE,” “Radial RMSE”), the coefficient of determination (columns “Linear R²,” “Radial R²”) for both linear and radial SVM regression models, and the sum of ranks of these performance metrics for both linear and radial SVM models (column “Sum of Ranks”) indicating overall performance (all column headers are highlighted in bold). The complete ranking table, including the ranking scores for unidentified metabolites, is provided in Supplementary Table 11.

### Biospecimen collection, quality control and sample accessioning

Blood plasma samples were collected for all study participants from the Luxembourg Parkinson’s Study and were submitted for liquid chromatography–mass spectrometry (LC-MS) metabolomics profiling using the Metabolon untargeted global metabolomics screening platform (www.metabolon.com). Plasma was recovered from ethylenediaminetetraacetic acid (EDTA) coated blood collection tubes (Becton Dickinson Ref. 367525) by centrifugation at 2000 g for 10 minutes at room temperature. The entire plasma supernatant was transferred to a 15 ml polypropylene conical mixing tube (VWR Ref. 525-0401) taking care not to aspirate from the buffy coat layer between the plasma and the erythrocytes, either by automated script on a TECAN liquid handling platform (Freedom EVO, TECAN) or manually with sterile serological pipettes. The collected supernatant was homogenized by repeated aspiration and dispense cycles and then pipetted into 220 µl aliquots into barcode labeled screw-cap cryovials of approximately 700 µl capacity (Thermo MATRIX 0.5 ml, Thermo-Fisher ref.3744 or FluidX 0.7 ml, Azenta ref, 68-0703-10) and frozen and stored at −80 °C in 96-position SBS-format lockable racks. Up to 12 aliquots of 220 µl could be obtained from each 10 ml EDTA blood collection tube.

To detect and filter blood samples of low quality, the Integrated Biobank of Luxembourg (IBBL), which processed the study samples, performed the following assays as part of its routine quality control: 1) Complete blood cell counting using an ABX Micros 60 Hematology Analyzer on a small aliquot of the whole blood before centrifugation, providing the following 6 parameters: White blood cell count (WBC), red blood cell count (RBC), hemoglobin (HGB), mean corpuscular volume (MCV), hematocrit (HCT), C-reactive protein (CRP); 2) HIL (hemolysis, icterus and lipemia) indices are tested using a COBAS Integra SI4 assay on the recovered pooled and mixed plasma prior to aliquoting. In addition, IBBL conducts an annual program of continuous quality control monitoring of all its processing methods, which includes assays performed on derivatives of EDTA blood samples collected and processed under the same conditions throughout the year specifically for IBBL’s annual quality control (QC) program. Assays performed on the plasma derivatives include the following: 1) Platelet count; 2) Interleukin-16 (IL-16) concentration; 3) HIL (hemolysis, icterus and lipemia) indices are tested using a COBAS Integra SI4 assay on the recovered pooled and mixed plasma prior to aliquoting; 4) Consistency of aliquot volumes (automated process). Hemolyzed blood samples and samples highlighted as abnormal or problematic by instrument flags or measurements for the above readouts are not included in any further analyses, such as the metabolomics profiling analyses conducted for our study. Further quality control analyses were performed as part of the metabolomics profiling (see sub-section on “Quality analysis and quality controls (QC)” in the section on “Metabolomics sample processing”).

Following receipt of the samples at Metabolon on dry ice, the samples were inventoried and immediately stored at −80 °C. Each sample received was accessioned into the Metabolon Laboratory Information Management System (LIMS) and assigned a unique identifier associated with the source identifier only. This identifier was used to track all sample handling, tasks, and results. All samples were maintained at −80^o^C until processed.

### Metabolomics sample processing

The processing of the metabolomics samples, including sample preparation, profiling, quality analyses and quality controls (QC), followed the standard procedure by Metabolon as described below (extracted from the documentation provided by Metabolon along with the experimental data).

### Sample preparation for metabolomics

Samples were prepared using the automated MicroLab STAR® system from Hamilton Company. Several recovery standards were added prior to the first step in the extraction process for QC purposes. To remove protein, dissociate small molecules bound to protein or trapped in the precipitated protein matrix, and to recover chemically diverse metabolites, proteins were precipitated with methanol under vigorous shaking for 2 min (GenoGrinder 2000 by the supplier Glen Mills) followed by centrifugation. The resulting extract was divided into five fractions: two for analysis by two separate reverse phase Ultrahigh Performance Liquid Chromatography-Tandem Mass Spectroscopy (RP/UPLC-MS/MS) methods with positive ion mode electrospray ionization (ESI), one for analysis by RP/UPLC-MS/MS with negative ion mode ESI, one for analysis by HILIC/UPLC-MS/MS with negative ion mode ESI, and one sample was reserved for backup. Samples were placed briefly on a TurboVap® (Zymark) to remove the organic solvent. The sample extracts were desolvated under nitrogen and stored at −80 °C before preparation for analysis. In total, the samples underwent two freeze-thaw cycles during the entire course of the study, including one for the experimental processing at Metabolon. The aliquots were always processed in parallel for the samples, i.e., all samples experienced consistent handling, undergoing the same number of freeze-thaw cycles.

### Metabolomics profiling

The metabolomics measurements for all samples were conducted using Ultra-Performance Liquid Chromatography-Tandem Mass Spectroscopy (UPLC-MS/MS). All methods used a Waters ACQUITY ultra-performance liquid chromatography (UPLC) and a Thermo Scientific Q-Exactive high resolution/accurate mass spectrometer interfaced with a heated electrospray ionization (HESI-II) source and Orbitrap mass analyzer operated at 35,000 mass resolution. The sample extract was dried, then reconstituted in solvents compatible with each of the four methods. Each reconstitution solvent contained a series of standards at fixed concentrations to ensure injection and chromatographic consistency. One aliquot was analyzed using acidic positive ion conditions, chromatographically optimized for more hydrophilic compounds. In this method, the extract was gradient eluted from a C18 column (Waters UPLC BEH C18-2.1 × 100 mm, 1.7 µm) using water and methanol, containing 0.05% perfluoropentanoic acid (PFPA) and 0.1% formic acid (FA). Another aliquot was also analyzed using acidic positive ion conditions, however it was chromatographically optimized for more hydrophobic compounds. In this method, the extract was gradient eluted from the same aforementioned C18 column using methanol, acetonitrile, water, 0.05% PFPA and 0.01% FA and was operated at an overall higher organic content. Another aliquot was analyzed using basic negative ion optimized conditions using a separate dedicated C18 column. The basic extracts were gradient eluted from the column using methanol and water, however with 6.5 mM Ammonium Bicarbonate at pH 8. The fourth aliquot was analyzed via negative ionization following elution from a HILIC column (Waters UPLC BEH Amide 2.1 × 150 mm, 1.7 µm) using a gradient consisting of water and acetonitrile with 10 mM Ammonium Formate, pH 10.8. The MS analysis alternated between MS and data-dependent MS^n^ scans using dynamic exclusion. The scan range varied between methods but covered 70–1000 m/z. Raw data files were archived, extracted and further processed as described below.

### Quality analysis and quality controls (QC)

Multiple types of controls were analyzed in concert with the experimental samples: a pooled matrix sample generated by taking a small volume of each experimental sample served as a technical replicate throughout the data set; extracted water samples served as process blanks, aliquots of solvents used in extraction served as solvent blanks; and QC standards that were carefully chosen not to interfere with the measurement of endogenous compounds were spiked into every analyzed sample, allowed instrument performance monitoring and aided chromatographic alignment (Supplementary Tables 1 and 2, and Supplementary Fig. 1 describe these Metabolon QC samples and standards).

The use of ultrapure water as a blank sample is part of the standard operating procedures by Metabolon to assess the contribution to compound signals from the analytical procedures. This same process is used for all sample types processed at Metabolon including those not involving blood/plasma. Water is used instead of phosphate-buffered saline (PBS), because while PBS has comparable osmolarity and buffering capacity to plasma, it also contains salts and other components that could potentially interfere with the detection and quantification of metabolites. The use of water ensures that the blank does not introduce any extraneous peaks or signals into the mass spectrometry data, allowing for clearer interpretation of the results from the experimental samples. In addition, the use of water provides a “clean” background against which the performance of quality control (QC) standards can be evaluated. As the blank sample is also used to assess any contamination or carry-over effects during the sample preparation and analysis process, the use of water helps to identify any such issues more clearly than using a more complex matrix such as PBS.

Instrument variability was determined by calculating the median relative standard deviation (RSD) for the standards that were added to each sample prior to injection into the mass spectrometers. Overall process variability was checked by calculating the median RSD for all endogenous metabolites (i.e., non-instrument standards) present in 100% of the pooled matrix samples (see the median RSD values for instrument variability and process variability in Supplementary Table 3). Experimental samples were randomized across the batches, and with QC samples spaced evenly among the injections.

### Data quality control, filtering, and pre-processing for the metabolomics data

The quality control (QC) procedures encompassed the analysis of multiple controls alongside the experimental samples, including process blanks, solvent blanks, technical replicates, and carefully selected QC standards to monitor instrument performance and assist in chromatographic alignment (Supplementary Tables 1 and 2, and Supplementary Fig. 1 describe these Metabolon QC samples and standards; details on the quality analyses are described in the Supplementary Material section “Metabolomics sample processing - Quality analysis and quality controls”). Additionally, median relative standard deviations (RSD) were calculated for both instrument and process variability to ensure accurate and consistent results (see Supplementary Table 3 and Supplementary Fig. 2).

The raw metabolomics data was pre-processed to obtain metabolite abundances in the form of log-transformed, batch normalized and imputed peak-area data (i.e., total ion counts, which represent the integrated area-under-the-curve). Experimental samples were randomized across the batches, and with QC samples spaced evenly among the injections. The batch normalization was performed so that for each metabolite, the raw values for the samples were divided by the median in each instrument batch so that each batch and metabolite has a median of one. For every metabolite, the minimum value across the batches for the median-scaled data was used to impute the missing values (limitations associated with missing values and the rationale for the imputation approach are discussed in the section on “Study limitations”). The batch-normalized and imputed data was transformed using the natural logarithm. This was motivated by a comparison of average density estimation plots of the peak-area data before and after log transformation, suggesting that the log-transformed data better follows a normal distribution (see Supplementary Fig. 3). The final metabolomics dataset covered a total of 1490 biochemicals, covering 1207 compounds of known identity and 283 compounds of unknown structural identity. A complete list of these compounds, including information on their public database IDs, chemical properties, and associated biochemical pathways, is provided in Supplementary Table 4.

### Transcriptomics data

For the PD case-control brain transcriptomics data analyzed in this study^85^, pre-processed data was obtained from the database Gene Expression Omnibus (GEO, ID: GSE8397). The used samples are from the lateral *substantia nigra* midbrain region, covering 9 PD patients and 5 controls, obtained in the GSE Series Matrix file format and analyzed at the log scale.

### Statistical analyses of the metabolomics data

A detailed reporting form, providing standardized information on the metabolomics data by integrating relevant recommendations from the “Core Information for Metabolomics Reporting (CIMR)” by the Metabolomics Standards Initiative^86^ and the Co-ordination of Standards in Metabolomics^87^ is provided as a Supplementary Material. To avoid that treatment effects resulting from standard drug therapy for PD patients involving medications containing the active compound levodopa (L-DOPA) affect the metabolomics data analysis, we first focused on the subset of de novo PD patients. A differential abundance analysis comparing these 56 de novo patients against all 590 controls, was performed using the empirical Bayes moderated t-statistic^88^ as implemented in the R software package *limma* (v3.52.2, RRID:SCR_010943)^89^, adjusting for age and sex as confounders. The resulting *p* values were corrected for multiple hypothesis testing according to the Benjamini and Hochberg method^90^. Next, the differential abundance analysis was repeated for the entire cohort of PD patients, i.e., comparing 549 patients against 590 controls, using the same statistical approach but including the abundance measurements for the L-DOPA metabolite 3-O-Methyldopa (3-OMD, 3-methoxytyrosine) as an additional covariate to adjust for dopaminergic treatment effects (L-DOPA itself was not covered among the measured metabolites, see section on “Study limitations”). Prior to all differential abundance analyses, metabolite features with zero variance across the considered samples were removed. Since L-DOPA medication has a pronounced effect on blood metabolite measurements, we additionally filtered out all metabolites from the data with a minimum absolute Spearman correlation of 0.2 to the 3-OMD abundances prior to the differential analysis. We note that weak indirect treatment effects may persist in the data after these filtering and adjustment steps. Therefore, we mainly rely on the prior de novo patient vs. control comparison to assess treatment-independent effects.

By conducting the differential analysis for de novo patients and for the entire cohort of PD patients, with the described additional filtering and covariate adjustments, we identified the set of shared significant differential metabolites with the same direction of the change in these two analyses as the set of high-confidence PD-associated metabolites, whose alterations are both independent of treatment effects (as confirmed by the de novo differential analysis) and robust across a large sample size (as confirmed by the analysis of the entire PD dataset). While the analysis of the entire PD cohort (including de novo patients and treated patients) in addition to the de novo patient-specific analysis allowed us to maximize statistical power to detect PD-associated changes, we also performed a dedicated analysis for treated patients only, using the same filtering and adjustment steps as for the entire PD dataset, to better distinguish changes in treated from treatment-naïve patients.

In addition, we used available clinical measurements of motor impairment severity, quantified by the Movement Disorder Society‐Sponsored Revision of the Unified Parkinson’s Disease Rating Scale (MDS‐UPDRS) Part III Motor Scores^91^, to build linear models for testing associations between metabolite profiles and the severity of motor symptoms. This analysis was conducted using the data from all patients, adjusting for sex, age, and L-DOPA medication, and correcting the significance scores for multiple hypothesis testing in the same manner as for the differential abundance analysis.

### Statistical analyses of transcriptomics data

The gene expression data was analyzed by comparing PD vs. control samples from the lateral *substantia nigra* brain region using the same implementation of the empirical Bayes moderated t-statistic as for the metabolomics data. We again adjusted the analysis for the available confounding factor variables age and sex and performed multiple testing corrections for the *p* values using the Benjamini and Hochberg method. MDS‐UPDRS motor scores and data on dopaminergic treatment were not available for the subjects covered for the transcriptomics profiling, and therefore the comparison of metabolomics and transcriptomics data focused on the case-control analyses and shared network alterations, confirming the treatment-independence using the metabolomics data (see section on “Pathway and network analyses” below).

All statistical analyses and associated volcano plot and dot plot visualizations were implemented in the R statistical programming software (version 4.2.0)^92^. The results were computed on a physical machine (CentOS 7.9.2009, Kernel: 3.10.0-1160.25.1.el7.x86_64).

### Pathway and network analyses

Pathway enrichment analyses for the metabolomics data were conducted using the MetaboAnalyst software^54^. As annotation data resources, we used cellular pathway definitions from the database KEGG^53^ and metabolite sets representing chemical structure classes directly from MetaboAnalyst^54^. The complete set of identifiable, experimentally profiled metabolites was used as a reference metabolome for the pathway analyses, and from the pathway annotation databases only metabolite sets covering at least 5 metabolites were considered. To obtain a comprehensive coverage of pathways enriched in putative PD-associated changes, we tested the over-representation of both metabolites with false-discovery rate (FDR)-adjusted and nominal significance (*p* <= 0.05) in pathways and metabolite sets from these databases.

Next, to investigate the network relationships between metabolites and enzymes undergoing coordinated changes in PD, integrated network analyses of the differential metabolomics and transcriptomics data statistics were implemented using the “Build Network” analysis workflow in the GeneGo MetaCore™ software with a focus on human molecular interaction data (filtered to species *homo sapiens*). All resulting *p* values for the pathway and network analyses were adjusted according to Benjamini and Hochberg^90^.

### Machine learning analyses

To obtain a first estimate of the utility of the metabolomics data for predictive modeling of disease outcomes, we performed machine learning (ML) analyses for PD vs. control diagnostic discrimination (classification analysis) and UPDRS III total motor score prediction (regression analysis). To avoid the strong confounding effects of dopaminergic medication in the data from treated patients, only data from de novo patients and controls were used for the PD vs. control classification analysis. By contrast, the regression analysis of the UPDRS III total motor score as a measure of disease severity was performed only for treated patients, excluding controls and de novo patients, because monitoring of motor performance is arguably most relevant for the majority of treated patients who are no longer in the initial stages of the disease. The aim of this specific analysis was to investigate whether metabolomics data could serve as a surrogate marker for UPDRS III motor assessments, potentially providing a means to replace or complement some of the routine assessments in the clinic with molecular measurements that may be less time consuming and burdensome for patients. Importantly, although regression models may be influenced strongly by the impact of dopaminergic medication on the metabolomics data, they may still be of practical use if they can accurately predict UPDRS III motor performance, e.g., to monitor the effects of medication.

For the ML analyses, the data was divided into training and testing sets in a 66:34 ratio, using a predetermined random seed to ensure reproducibility. Support Vector Machines (SVMs) were employed for the model building considering both a linear kernel and a radial basis function (RBF) kernel to detect both linear and non-linear predictive patterns. The R software package *e1071* was used to train and apply the SVM models (https://cran.r-project.org/package=e1071, version 1.7-11). To optimize hyperparameters, a grid search was conducted on the training data within a 5-fold cross-validation framework. A key aspect of our methodology was the selection of the least complex model (with the lowest value of the regularization parameter C in the SVM) that was within one standard deviation of the best-performing model (in terms of cross-validated area under the Receiver Operating Characteristic Curve (AUC) for the classification analysis, and in terms of the root mean squared error (RMSE) for the regression analysis). This approach is grounded in the principle of preferring simpler models with similar predictive ability to avoid overfitting and increase generalizability. Once the most suitable models in terms of this performance/complexity trade-off criterion were identified, they were retrained with the selected hyperparameters on the entire training set. The retrained models were then applied to the test set to further evaluate their performance on independent samples. To assess the discriminative power of individual features, we conducted the above ML and cross-validation analyses for each metabolite feature in isolation. This involved training SVM models for each feature and assessing the predictive performance for both the classification and the regression analysis. Feature rankings were then consolidated by computing the average AUC for the classification analysis, and respectively, the sum of feature ranks for the regression analysis, for both linear kernel and RBF kernel SVMs for the training set cross-validation and the independent test set evaluation. This composite ranking provided a summarized view of feature importance to distinguish the most informative features for both classification and regression tasks.

### Reporting summary

Further information on research design is available in the Nature Research Reporting Summary linked to this article.

### Supplementary information

Supplementary Materials (Sample processing details, Suppl. Figure 1, Suppl. Tables 1, 2, 6 and 7)

Reporting Summary

Supplementary Table 4

Supplementary Table 5

Supplementary Table 6

Supplementary Table 7

Supplementary Table 8

Supplementary Table 11

Supplementary Table 12

Supplementary Table 13

## Data Availability

Public transcriptomics data was obtained from the GEO database (ID: GSE8397). Spreadsheet versions of the metabolite list and larger ranking tables (Supplementary Tables 4–7, 11 and 12) have been made available on the public GitLab repository (https://gitlab.lcsb.uni.lu/bds/pd_metabolomics). The metabolomics dataset for this manuscript is not publicly available as it is linked to the Luxembourg Parkinson’s Study and its internal regulations. Any requests for accessing the dataset can be directed to request.ncer-pd@uni.lu.

## Code availability

The statistical analyses were implemented in R (v4.2.0), and the code is available under the MIT license in the following GitLab repository: https://gitlab.lcsb.uni.lu/bds/pd_metabolomics.

## References

[1] Magdalinou, N. & Morris, H. R. Clinical features and differential diagnosis of Parkinson’s disease. in *Movement Disorders Curricula* 103–115 (Springer Vienna, 2017).

[2] Yang T (2017). 131I-MIBG myocardial scintigraphy for differentiation of Parkinson’s disease from multiple system atrophy or essential tremor in Chinese. Popul. J. Neurol. Sci..

[3] Delenclos M, Jones DR, McLean PJ, Uitti RJ (2016). Biomarkers in Parkinson’s disease: Advances and strategies. Parkinsonism Relat. Disord..

[4] Blauwendraat C, Nalls MA, Singleton AB (2020). The genetic architecture of Parkinson’s disease. Lancet Neurol..

[5] Dixit A, Mehta R, Singh AK (2019). Proteomics in Human Parkinson’s Disease: Present scenario and future directions. Cell. Mol. Neurobiol..

[6] Li X, Fan X, Yang H, Liu Y (2022). Review of metabolomics-based biomarker research for Parkinson’s disease. Mol. Neurobiol..

[7] Bogdanov M (2008). Metabolomic profiling to develop blood biomarkers for Parkinson’s disease. Brain.

[8] Chelliah SS, Bhuvanendran S, Magalingam KB, Kamarudin MNA, Radhakrishnan AK (2022). Identification of blood-based biomarkers for diagnosis and prognosis of Parkinson’s disease: A systematic review of proteomics studies. Ageing Res. Rev..

[9] Tönges L (2022). Blood-based biomarker in Parkinson’s disease: potential for future applications in clinical research and practice. J. Neural Transm..

[10] Hipp G (2018). The Luxembourg Parkinson’s Study: A comprehensive approach for stratification and early diagnosis. Front. Aging Neurosci..

[11] Gao X (2007). Prospective study of dietary pattern and risk of Parkinson disease. Am. J. Clin. Nutr..

[12] Baroni L (2011). Pilot dietary study with normoproteic protein-redistributed plant-food diet and motor performance in patients with Parkinson’s disease. Nutr. Neurosci..

[13] Mischley LK, Lau RC, Bennett RD (2017). Role of diet and nutritional supplements in Parkinson’s disease progression. Oxid. Med. Cell. Longev..

[14] Wishart DS (2022). HMDB 5.0: the Human Metabolome Database for 2022. Nucleic Acids Res..

[15] Visconti, A. et al. Interplay between the human gut microbiome and host metabolism. *bioRxiv* 561787 (2019) 10.1101/561787. 10.1038/s41467-019-12476-z PMC6776654 31582752

[16] Burté F (2017). metabolic profiling of Parkinson’s disease and mild cognitive impairment. Mov. Disord..

[17] Luce M (2018). Is 3-Carboxy-4-methyl-5-propyl-2-furanpropionate (CMPF) a Clinically Relevant Uremic Toxin in Haemodialysis Patients?. Toxins.

[18] Zhu Y, Wang P, Sha W, Sang S (2016). Urinary biomarkers of whole grain wheat intake identified by non-targeted and targeted metabolomics approaches. Sci. Rep..

[19] D’Silva S, Haider SJ, Phizicky EM (2011). A domain of the actin binding protein Abp140 is the yeast methyltransferase responsible for 3-methylcytidine modification in the tRNA anti-codon loop. RNA.

[20] Bohnsack KE, Kleiber N, Lemus-Diaz N, Bohnsack MT (2022). Roles and dynamics of 3-methylcytidine in cellular RNAs. Trends Biochem. Sci..

[21] Zhang X (2020). Small RNA modifications in Alzheimer’s disease. Neurobiol. Dis..

[22] Bednářová A (2017). Lost in translation: defects in transfer RNA modifications and neurological disorders. Front. Mol. Neurosci..

[23] Saiki S (2017). Decreased long-chain acylcarnitines from insufficient β-oxidation as potential early diagnostic markers for Parkinson’s disease. Sci. Rep..

[24] Ueno S-I (2018). Zonisamide administration improves fatty acid β-oxidation in Parkinson’s disease. Cells.

[25] Keshavarzian A (2015). Colonic bacterial composition in Parkinson’s disease. Mov. Disord..

[26] Hill-Burns EM (2017). Parkinson’s disease and Parkinson’s disease medications have distinct signatures of the gut microbiome. Mov. Disord..

[27] Petrov VA (2017). Analysis of gut microbiota in patients with Parkinson’s disease. Bull. Exp. Biol. Med..

[28] Unger MM (2016). Short chain fatty acids and gut microbiota differ between patients with Parkinson’s disease and age-matched controls. Parkinsonism Relat. Disord..

[29] Li W (2017). Structural changes of gut microbiota in Parkinson’s disease and its correlation with clinical features. Sci. China Life Sci..

[30] Scheperjans F (2015). Gut microbiota are related to Parkinson’s disease and clinical phenotype. Mov. Disord..

[31] Baert F (2021). Parkinson’s disease patients’ short chain fatty acids production capacity after in vitro fecal fiber fermentation. NPJ Parkinsons Dis..

[32] Zhang Y (2023). Sodium butyrate ameliorates gut dysfunction and motor deficits in a mouse model of Parkinson’s disease by regulating gut microbiota. Front. Aging Neurosci..

[33] Xu R-C (2022). Neuroprotective effects of sodium butyrate and monomethyl fumarate treatment through GPR109A modulation and intestinal barrier restoration on PD mice. Nutrients.

[34] Liu J (2017). Sodium butyrate exerts protective effect against Parkinson’s disease in mice via stimulation of glucagon like peptide-1. J. Neurol. Sci..

[35] Qiao C-M (2020). Sodium butyrate exacerbates Parkinson’s disease by aggravating neuroinflammation and colonic inflammation in MPTP-induced Mice model. Neurochem. Res..

[36] Alecu I, Bennett SAL (2019). Dysregulated lipid metabolism and its role in α-synucleinopathy in Parkinson’s disease. Front. Neurosci..

[37] Herrmann G, Jayamani E, Mai G, Buckel W (2008). Energy conservation via electron-transferring flavoprotein in anaerobic bacteria. J. Bacteriol..

[38] Siemers ER, Shekhar A, Quaid K, Dickson H (1993). Anxiety and motor performance in Parkinson’s disease. Mov. Disord..

[39] Link KP (1945). & Others. The anticoagulant 3, 3’-methylenebis (4-hydroxycoumarin). Federation Proc. Federation Am. Societies Exp. Biol..

[40] Sharifi-Rad J (2021). Natural coumarins: exploring the pharmacological complexity and underlying molecular mechanisms. Oxid. Med. Cell. Longev..

[41] Tao D (2019). Discovery of coumarin Mannich base derivatives as multifunctional agents against monoamine oxidase B and neuroinflammation for the treatment of Parkinson’s disease. Eur. J. Med. Chem..

[42] Kanehisa M, Goto S (2000). KEGG: kyoto encyclopedia of genes and genomes. Nucleic Acids Res.

[43] Pang Z (2021). MetaboAnalyst 5.0: narrowing the gap between raw spectra and functional insights. Nucleic Acids Res.

[44] Hatano T, Saiki S, Okuzumi A, Mohney RP, Hattori N (2016). Identification of novel biomarkers for Parkinson’s disease by metabolomic technologies. J. Neurol. Neurosurg. Psychiatry.

[45] Deleu D, Jacob P, Chand P, Sarre S, Colwell A (2006). Effects of caffeine on levodopa pharmacokinetics and pharmacodynamics in Parkinson disease. Neurology.

[46] Karobath M, Díaz JL, Huttunen MO (1971). The effect of L-dopa on the concentrations of tryptophan, tyrosine and serotonin in rat brain. Eur. J. Pharmacol..

[47] Blokland A, Lieben C, Deutz NEP (2002). Anxiogenic and depressive-like effects, but no cognitive deficits, after repeated moderate tryptophan depletion in the rat. J. Psychopharmacol..

[48] Neumeister A (2003). Tryptophan depletion, serotonin, and depression: where do we stand?. Psychopharmacol. Bull..

[49] Fujimaki M (2018). Serum caffeine and metabolites are reliable biomarkers of early Parkinson disease. Neurology.

[50] Shi D (2021). The serum metabolome of COVID-19 patients is distinctive and predictive. Metabolism.

[51] Signorelli P, Conte C, Albi E (2021). The Multiple Roles of Sphingomyelin in Parkinson’s Disease. Biomolecules.

[52] Ikenaka K, Suzuki M, Mochizuki H, Nagai Y (2019). Lipids as Trans-Acting Effectors for α-Synuclein in the Pathogenesis of Parkinson’s Disease. Front. Neurosci..

[53] Massey LK, Roman-Smith H, Sutton RA (1993). Effect of dietary oxalate and calcium on urinary oxalate and risk of formation of calcium oxalate kidney stones. J. Am. Diet. Assoc..

[54] Casida JE (2014). Benomyl, aldehyde dehydrogenase, DOPAL, and the catecholaldehyde hypothesis for the pathogenesis of Parkinson’s disease. Chem. Res. Toxicol..

[55] Xicoy H, Brouwers JF, Wieringa B, Martens GJM (2020). Explorative combined lipid and transcriptomic profiling of substantia nigra and putamen in Parkinson’s disease. Cells.

[56] Spector R (1987). Hypoxanthine transport through the blood-brain barrier. Neurochem. Res..

[57] Doubrovin M (2003). Development of a new reporter gene system–dsRed/xanthine phosphoribosyltransferase-xanthine for molecular imaging of processes behind the intact blood-brain barrier. Mol. Imaging.

[58] Basile MS, Bramanti P, Mazzon E (2022). Inosine in Neurodegenerative Diseases: From the Bench to the Bedside. Molecules.

[59] Torres RJ, Puig JG (2007). Hypoxanthine-guanine phosophoribosyltransferase (HPRT) deficiency: Lesch-Nyhan syndrome. Orphanet J. Rare Dis..

[60] López Jiménez, M. et al. [Purine transport through the blood-brain barrier in hypoxanthine phosphoribosyltransferase deficiency]. Med. Clin. 92, 167–170 (1989). 2725104

[61] Johnson TA, Jinnah HA, Kamatani N (2019). Shortage of cellular ATP as a cause of diseases and strategies to enhance ATP. Front. Pharmacol..

[62] Winklhofer KF, Haass C (2010). Mitochondrial dysfunction in Parkinson’s disease. Biochim. Biophys. Acta.

[63] Vinokurov AY (2023). HPRT1 deficiency induces alteration of mitochondrial energy metabolism in the brain. Mol. Neurobiol..

[64] Li W-X (2020). Systematic metabolic analysis of potential target, therapeutic drug, diagnostic method and animal model applicability in three neurodegenerative diseases. Aging.

[65] Watanabe H (2020). Improved Parkinsons disease motor score in a single-arm open-label trial of febuxostat and inosine. Medicine.

[66] Jiang J, Piao X, Hu S, Gao J, Bao M (2020). LncRNA H19 diminishes dopaminergic neuron loss by mediating microRNA-301b-3p in Parkinson’s disease via the HPRT1-mediated Wnt/β-catenin signaling pathway. Aging.

[67] Sahoo S, Franzson L, Jonsson JJ, Thiele I (2012). A compendium of inborn errors of metabolism mapped onto the human metabolic network. Mol. Biosyst..

[68] Aarsland D (2021). Parkinson disease-associated cognitive impairment. Nat. Rev. Dis. Prim..

[69] LeWitt P, Schultz L, Auinger P, Lu M (2011). & Parkinson Study Group DATATOP Investigators. CSF xanthine, homovanillic acid, and their ratio as biomarkers of Parkinson’s disease. Brain Res.

[70] Janitschke D (2021). Methylxanthines and neurodegenerative diseases: an update. Nutrients.

[71] Cipriani S, Bakshi R, Schwarzschild MA (2014). Protection by inosine in a cellular model of Parkinson’s disease. Neuroscience.

[72] Bakshi R (2015). Neuroprotective effects of urate are mediated by augmenting astrocytic glutathione synthesis and release. Neurobiol. Dis..

[73] Isaacson SH, Betté S, Pahwa R (2022). Istradefylline for OFF episodes in Parkinson’s disease: A US perspective of common clinical scenarios. Degener. Neurol. Neuromuscul. Dis..

[74] Riederer P, Laux G (2011). MAO-inhibitors in Parkinson’s Disease. Exp. Neurobiol..

[75] Litvan I (2003). Movement disorders society scientific issues committee report: SIC Task Force appraisal of clinical diagnostic criteria for Parkinsonian disorders. Mov. Disord..

[76] Moran LB (2006). Whole genome expression profiling of the medial and lateral substantia nigra in Parkinson’s disease. Neurogenetics.

[77] Fiehn O (2007). The metabolomics standards initiative (MSI). Metabolomics.

[78] Salek RM (2015). COordination of Standards in MetabOlomicS (COSMOS): facilitating integrated metabolomics data access. Metabolomics.

[79] Smyth GK (2004). Linear models and empirical bayes methods for assessing differential expression in microarray experiments. Stat. Appl. Genet. Mol. Biol..

[80] Ritchie ME (2015). limma powers differential expression analyses for RNA-sequencing and microarray studies. Nucleic Acids Res.

[81] Benjamini Y, Hochberg Y (1995). Controlling the false discovery rate: a practical and powerful approach to multiple testing. J. R. Stat. Soc. Ser. B Stat. Methodol..

[82] Goetz CG (2008). Movement Disorder Society-sponsored revision of the Unified Parkinson’s Disease Rating Scale (MDS-UPDRS): scale presentation and clinimetric testing results. Mov. Disord..

[83] Core Team, R. RA Language and Environment for Statistical Computing. R Foundation for Statistical Computing, Vienna, Austria.-References. Scientific Research Publishing.

[84] Kostić DA (2015). Xanthine oxidase: Isolation, assays of activity, and inhibition. J. Chem..

[85] Gökçe Çokal B (2017). Serum glutathione peroxidase, xanthine oxidase, and superoxide dismutase activities and malondialdehyde levels in patients with Parkinson’s disease. Neurol. Sci..

[86] Wu G, Lupton JR, Turner ND, Fang Y-Z, Yang S (2004). Glutathione Metabolism and Its Implications for Health. J. Nutr..

[87] Xu K, Xu Y-H, Chen J-F, Schwarzschild MA (2010). Neuroprotection by caffeine: time course and role of its metabolites in the MPTP model of Parkinson’s disease. Neuroscience.

[88] Hong CT, Chan L, Bai C-H (2020). The effect of caffeine on the risk and progression of Parkinson’s disease: a meta-analysis. Nutrients.

[89] Kasabova-Angelova A (2020). Xanthine derivatives as agents affecting non-dopaminergic neuroprotection in Parkinson’s disease. Curr. Med. Chem..

[90] Müller T (2015). The safety of istradefylline for the treatment of Parkinson’s disease. Expert Opin. Drug Saf..

[91] Jost WH (2022). A critical appraisal of MAO-B inhibitors in the treatment of Parkinson’s disease. J. Neural Transm..

[92] Goldstein DS (2021). The catecholaldehyde hypothesis for the pathogenesis of catecholaminergic neurodegeneration: what we know and what we do not know. Int. J. Mol. Sci..

