# Supplementary material for: Comprehensive blood metabolomics profiling of Parkinson’s disease reveals coordinated alterations in xanthine metabolism
Source: NPJ Parkinsons Dis. 2024 Mar 19;10:68. doi: 10.1038/s41531-024-00671-9 (PMC10951366; doi:10.1038/s41531-024-00671-9)
Supplement: Supplementary file 1 — Supplementary Materials (Sample processing details, Suppl. Figure 1, Suppl. Tables 1, 2, 6 and 7) [file 41531_2024_671_MOESM1_ESM.pdf]

Supplementary Materials

OUTLINE

- Suppl. Tab. 1: Description of Metabolon Quality Control Samples - Page 2
- Suppl. Tab. 2: Description of Metabolon Quality Control Standards - Page 2
- Suppl. Fig. 1: Preparation of Client-Specific Technical Replicates - Page 2
- Suppl. Tab. 3: Instrument and Process Variability - Page 3
- Suppl. Fig. 2: Histogram of relative standard deviation values - Page 3
- Suppl. Fig. 3: Average density estimation plots - Page 4
- Legends for Supplementary Tables 4 to 8 (provided as external files) - Page 4
- Suppl. Tab. 9: Metabolite set enrichment analysis (chemical structure classes) - Page 5
- Suppl. Tab. 10: Pathway enrichment analysis (KEGG) - Page 5
- Legends for Supplementary Tables 11 to 12 (provided as external files) - Page 5
- Metabolomics data reporting form - Page 7

Type	Description	Purpose
MTRX	Large pool of human plasma maintained by Metabolon that has been characterized extensively.	Assure that all aspects of the Metabolon process are operating within specifications.
CMTRX	Pool created by taking a small aliquot from every customer sample.	Assess the effect of a non-plasma matrix on the Metabolon process and distinguish biological variability from process variability.
PRCS	Aliquot of ultra-pure water	Process Blank used to assess the contribution to compound signals from the process.
SOLV	Aliquot of solvents used in extraction.	Solvent Blank used to segregate contamination sources in the extraction.

Suppl. Tab. 1: Description of Metabolon quality control samples.

Type	Description	Purpose
RS	Recovery Standard	Assess variability and verify performance of extraction and instrumentation.
IS	Internal Standard	Assess variability and performance of instrument.

Suppl. Tab. 2: Description of Metabolon quality control standards.

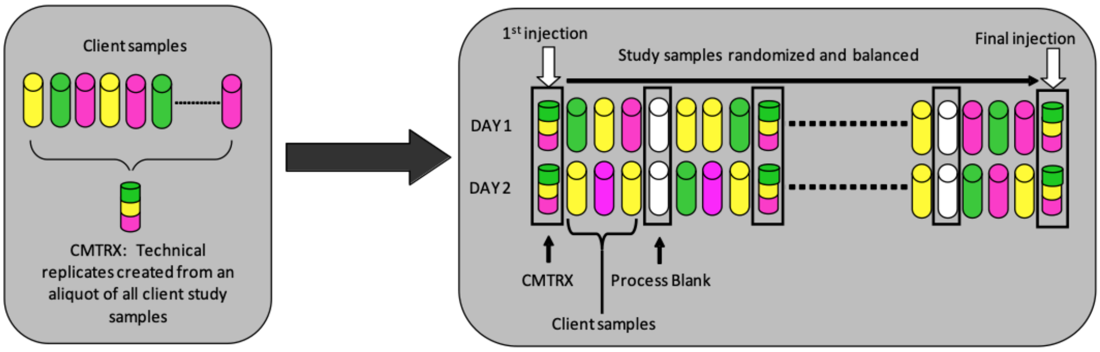

Suppl. Fig 1: Preparation of client-specific technical replicates. A small aliquot of each client sample (colored cylinders) is pooled to create a CMTRX technical replicate sample (multi-colored cylinder), which is then injected periodically throughout the platform run. Variability among consistently detected biochemicals can be used to calculate an estimate of overall process and platform variability.

QC Sample	Measurement	Median RSD
Internal Standards	Instrument Variability	7%
Endogenous Biochemicals	Total Process Variability	11%

Suppl. Tab. 3: Instrument and Process Variability. Instrument variability was determined by calculating the median relative standard deviation (RSD) for the internal standards that were added to each sample prior to injection into the mass spectrometers. Overall process variability was determined by calculating the median RSD for all endogenous metabolites (i.e., non-instrument standards) present in 100% of the MTXR7 technical replicates. Values for instrument and process variability meet Metabolon's acceptance criteria as shown in the table above.

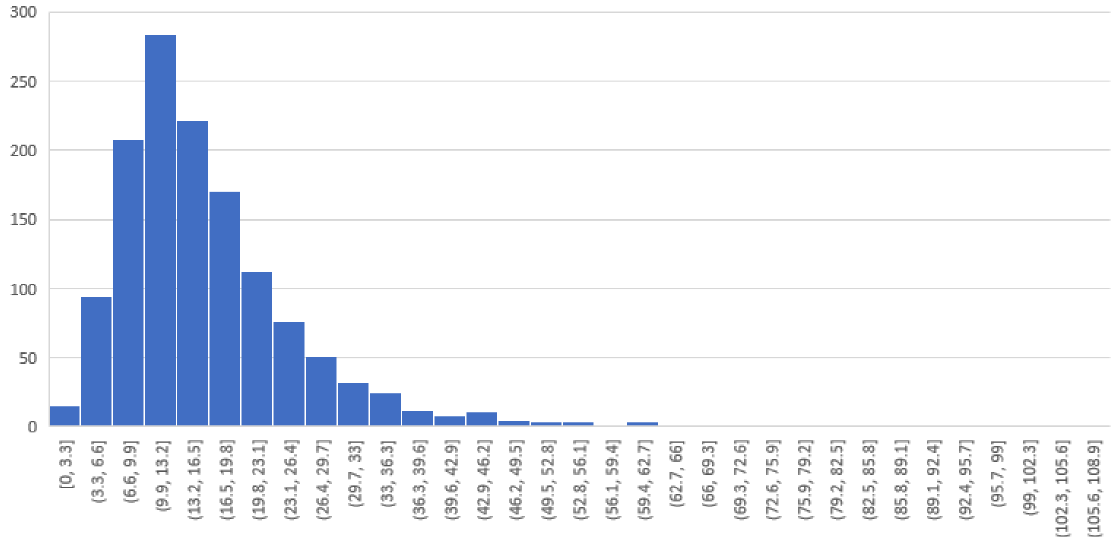

Suppl. Fig. 2: Histogram of relative standard deviation (RSD) values. A histogram has been generated which bins the RSD values for all molecules detected in MTRX7, including those not present in 100% of the MTXR7 technical replicates. The horizontal axis shows the bins of RSD values, the vertical axis the number of molecules within these bins). The calculated median RSD for this data is 14.

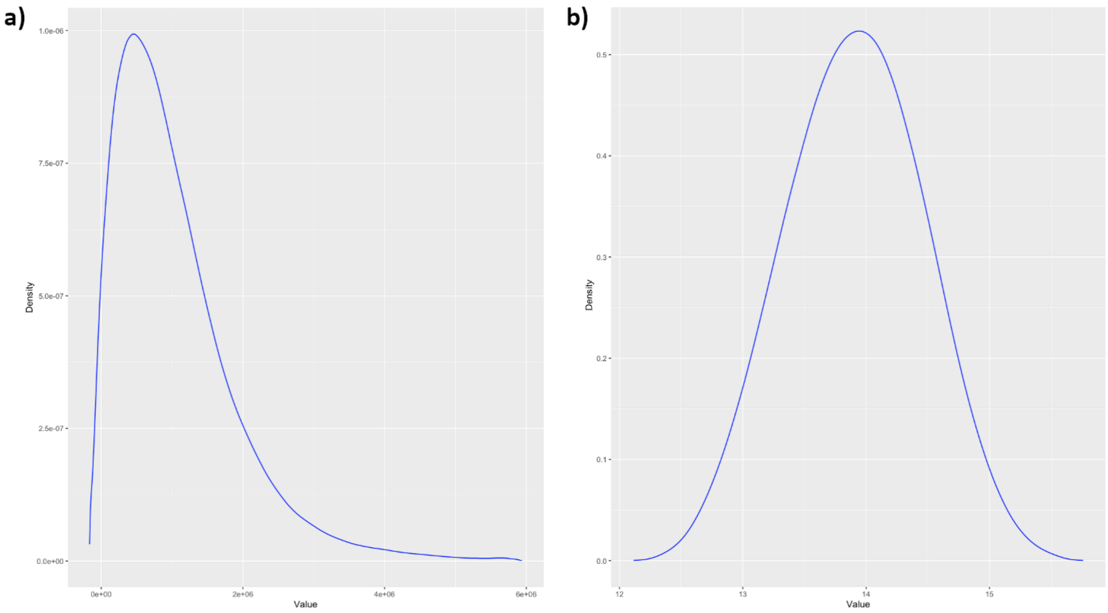

Suppl. Fig. 3: Average density estimation plots. a) Average density estimation plot of the peak area data prior to log transformation; b) Average density estimation plot of the peak area data after transformation using the natural log.

Suppl. Tab. 4: Complete list of studied metabolites. The complete list of studied metabolites, including public database IDs, chemical properties, and associated biochemical pathways (provided as a separate dataset file to enable further editing and processing by the reader).

Suppl. Tab. 5: Complete ranking table of metabolites in terms of differential abundance between *de novo* Parkinson's disease patients and controls. Statistics are adjusted for age and sex; logFC = log fold change between *de novo* Parkinson's disease and controls; P.Value = nominal p-value score; adj.P.Val = p-value adjusted for multiple hypothesis testing using the Benjamini-Hochberg approach (provided as a separate dataset file to enable further editing and processing by the reader).

Suppl. Tab. 6: Complete ranking table of metabolites in terms of differential abundance between all Parkinson's disease patients and controls in the cohort. Statistics are adjusted for age, sex and dopaminergic treatment effects; logFC = log fold change between Parkinson's disease and controls; P.Value = nominal p-value score; adj.P.Val = p-value adjusted for multiple hypothesis testing using the Benjamini-Hochberg approach(provided as a separate dataset file to enable further editing and processing by the reader).

Suppl. Tab. 7: Complete ranking table of metabolites in terms of differential abundance between Parkinson's disease patients who received dopaminergic medication and controls in the cohort. Statistics are adjusted for age, sex and dopaminergic treatment effects; logFC = log fold change between Parkinson's disease and controls; P.Value = nominal

p-value score; adj.P.Val = p-value adjusted for multiple hypothesis testing using the Benjamini-Hochberg approach (provided as a separate dataset file to enable further editing and processing by the reader).

Suppl. Table 8: Reference metabolome for the metabolite set enrichment analysis using the MetaboAnalyst software (Query = HMDB metabolite ID used for the query); Match = matched metabolite name recognized by MetaboAnalyst, NA otherwise; PubChem = PubChem identifier for mapped compounds; KEGG = KEGG identifier for mapped compounds, SMILES = SMILES code for mapped compounds; all mappings are derived from the MetaboAnalyst software). The table is provided as a separate dataset file to enable further editing and processing by the reader.

Metabolite Set	Total	Hits	Expect	P value	Holm P	FDR
Fatty Acids and Conjugates	96	25	14.5	0.00187	0.254	0.254
Fatty Acyls	10	4	1.51	0.0496	1.0	1.0

Suppl. Tab. 9: Metabolite set enrichment analysis results for chemical structure classes (main set) in MetaboAnalyst (showing all metabolites with a nominal *p*-value <= 0.05)

Metabolite Set	Total	Hits	Expect	P value	Holm P	FDR
Ubiquinone and other terpenoid-quinone biosynthesis	2	2	0.301	0.0226	1.0	0.803
Retinol metabolism	2	2	0.301	0.0226	1.0	0.803
Tyrosine metabolism	10	4	1.51	0.0496	1.0	0.803

Suppl. Tab. 10: Pathway enrichment analysis results for the KEGG database (showing all metabolites with a nominal *p*-value <= 0.05)

Suppl. Table 11: Ranking table of metabolite features in terms of their estimated predictive value for supervised sample classification of de novo PD and control samples, showing each metabolite feature (column Metabolite), the Area Under the ROC Curve for the training set (columns Train_Linear_AUC, Train_Radial_AUC) and for the test set (columns Test_Linear_AUC, Test_Radial_AUC) for both linear and radial Support Vector Machine (SVM) classifiers. The table is provided as a separate dataset file to enable further editing and processing by the reader.

Suppl. Table 12: Ranking table of metabolite features in terms of their estimated predictive value for regression analysis of UPDRS III total motor scores, displaying each metabolite feature (column Metabolite), the Mean Absolute Error (columns Linear_MAE, Radial_MAE), the coefficient of determination (columns Linear_R^2, Radial_R^2) for both linear and radial SVM regression models, their respective ranks (Linear_RMSE_Rank, Linear_R^2_Rank, Radial_MAE_Rank, Radial_R^2_Rank), and the total rank score (Sum_of_Ranks) indicating overall performance. The ranks for the Root Mean Square Error (RMSE) are sorted by increasing RMSE values, the ranks for the coefficient of determination (R^2) are sorted by

decreasing R^2 values. Rows are sorted by the sum of these ranks (column Sum_of_Ranks). The table is provided as a separate dataset file to enable further editing and processing by the reader.

Suppl. Table 13: Metadata for the metabolomics samples from the Luxembourg Parkinson's Study, including samples from patients receiving dopaminergic treatment (TREATED_PD), de novo patients (DENOVO_PD), controls (CONTROLS), and samples from a second study on longitudinal PD and atypical forms of parkinsonism, that were measured in the same batches, and are being prepared for separate publication (SECOND_STUDY). NR = Sample number and processing order; ND_CODE = subject pseudonym code; SAMPLE_ID = blood sample identifier; KIT_ID = blood collection tube identifier; SAMPLE_NAME = sample name used for metabolomics profiling of the Luxembourg Parkinson's Study; IDENTIFIER = numeric sample identifier for the metabolomics profiling; NEG, POLAR, POS.EARLY, POS.LATE = batch identifiers for a given experimental platform arm (NEG = Negative Ionization Mode, POLAR = Polar Compounds, POS.EARLY = Positive Ionization Mode, Early Retention Time, POS. LATE. Negative Ionizatin Mode, Late Retention Time); SAMPLE_GROUP = sample groups reflecting the different donor conditions and studies (TREATED_PD, DENOVO_PD, CONTROLS, SECOND_STUDY).

METABOLOMICS DATA REPORTING FORM

This form provides standardized information on the metabolomics study, integrating relevant recommendations from the "Core Information for Metabolomics Reporting (CIMR)" by the Metabolomics Standards Initiative (MSI; Fiehn et al., Metabolomics, 2007) and the Co-ordination of Standards in Metabolomics (COSMOS; Salek et al., Metabolomics, 2015).
Full details for the experimental metabolomics approach by the company Metabolon, which performed the measurements, are available in: Ford L, Kennedy AD, Goodman KD, Pappan KL, Evans AM, Miller LAD, Wulff JE, Wiggs BR, Lennon JJ, Elsea S, Toal DR. Precision of a Clinical Metabolomics Profiling Platform for Use in the Identification of Inborn Errors of Metabolism. J Appl Lab Med. 2020 Mar 1;5(2):342-356 (doi: 10.1093/jalm/jfz026, PMID: 32445384).

STUDY INFORMATION

- Study title: Comprehensive blood metabolomics profiling of Parkinson's disease reveals coordinated alterations in xanthine metabolism

- Authors/investigators: Elisa Gómez de Lope, Rebecca Ting Jiin Loo, Armin Rauschenberger, Muhammad Ali, Lukas Pavelka, Tainá M Marques, Clarissa P C Gomes, Rejko Krüger, Enrico Glaab on behalf of the NCER-PD Consortium

- Affiliations: Luxembourg Centre for Systems Biomedicine, University of Luxembourg; Luxembourg Institute of Health; Translational Neuroscience, University of Luxembourg; Parkinson's Research Clinic, Centre Hospitalier de Luxembourg

- Contact information for corresponding author: enrico.glaab@uni.lu

- Reporting date: 19.11.23

ETHICS APPROVAL

- Was ethical approval obtained for the study? Yes

- Name of ethics committee that approved the study: University of Luxembourg Ethics Review Panel (ref. ERP 18-042). All study subjects signed a written informed consent. The study was approved by the National Ethics Board in Luxembourg (CNER Ref: 201407/13) and complied with the Declaration of Helsinki. The Luxembourg Parkinson's study was registered in ClinicalTrials.gov under NCT05266872.

STUDY DESIGN

- Case definition and inclusion/exclusion criteria: Inclusion of diagnosed PD patients according to United Kingdom Parkinson's Disease Society Brain Bank (UKPDSBB) criteria. Exclusion criteria also follow UKPDSBB specifications.

- Control definition and inclusion/exclusion criteria: (i) age over 18 years, (ii) no evidence of neurodegenerative disorder; (iii) no active cancer, (iv) no pregnancy.

- Total number of cases and controls: 546 PD patients, 590 controls

- Case matching criteria: Age and sex adjusted during differential abundance analysis for *de novo* PD vs. control; case-control analyses involving patients who had received dopaminergic medication additionally include adjustments for the L-DOPA metabolite 3-O-Methyldopa (3-OMD)

- Randomization procedures: To adjust for potential confounding variables, we conducted a multivariate analysis using linear modeling techniques provided by the 'limma' package in R (see Methods section in the main manuscript).

STUDY POPULATION CHARACTERISTICS

- Geographical location of recruitment: Luxembourg and surrounding area defined as Greater Region.

- Age range, mean and standard deviation for cases and controls: PD patients: 65.9 ± 10.7 years; *de novo* PD patients: 67.2 ± 11.4 years; Controls: 61.7 ± 11.7 years

- Sex distribution for cases and controls: PD patients - 188 female, 358 male; *de novo* PD patients - 14 female, 42 male; Controls - 206 female, 384 male

- MDS-UPDRS III: PD patients - 32.7 ± 14.5; *de novo* PD - 32.3 ± 14.2; Controls - 3.69 ± 5.0

- Hoehn & Yahr stage: PD patients - 2.1 ± 0.7; *de novo* PD - 1.8 ± 0.57

- Disease duration since initial symptom (years): PD patients - 12.3 ± 7.3; *de novo* PD - 6.68 ± 5.4

- Body Mass Index (kg/m^2): PD patients - 27.5 ± 4.8; *de novo* PD - 28.2 ± 4.8; Controls - 27.6 ± 4.8

- Montreal Cognitive Assessment (MoCA): PD patients - 25.4 ± 3.2; *de novo* PD

- 24.9 ± 3.3; Controls - 26.9 ± 2.6

- SCales for Outcomes in PArkinson's disease - Autonomic Dysfunction (SCOPA-AUT): PD patients - 14.4 ± 8.0; *de novo* PD - 8.9 ± 5.3; Controls - 7.3 ± 5.6

- Dietary restrictions / fasting : none

- Relevant background information: Assessment of diabetes, hypercholesterolemia, cancer history status (see Table 1 in the main manuscript for further details)

SAMPLING PROTOCOL

- Protocol Title: Blood plasma sampling

- Source: Participants in the Luxembourg Parkinson's Study (Hipp et al., Front. Aging Neurosci., 2018)

- Organism: Homo sapiens sapiens

- Organism part: Blood plasma

- Factor description: PD patients vs. controls. Subsets - *de novo* PD patients and treated PD patients.

- Replicate sampling and analyses: 546 PD patients, 590 controls. 56 *de novo* PD subset.

- Location of collection: Nationwide in Luxembourg

- Time of collection: Not restricted, samples collected at various times throughout the day during routine clinical visits

- Volume collected: 10 ml (in EDTA tubes)

- Tissue / body fluid harvesting method: Venipuncture into Ethylenediaminetetraacetic acid (EDTA) tubes, centrifugation, transfer of plasma to cryovials

- Time from separation to freezing: Not specified

- Storage conditions and aliquoting: Stored at -80°C in aliquots until analysis

- Relocation or shipping info: Shipped on dry ice to the metabolomics service provider (Metabolon) for analysis

EXTRACTION PROTOCOL

- Protocol Title: Metabolon standard metabolite extraction
- Instrument: Hamilton ML STAR® MicroLab system
- Extraction solvent(s): Methanol
- Extract storage: Kept frozen at -80°C until analysis
- Sample prep for chromatography: Dried extract resuspended in solvents compatible with each analysis method (methanol, water)
- Other details: Proteins precipitated with methanol, vortex mixing and centrifugation. Extract divided into aliquots for analysis by RP/UPLC-MS/MS positive mode (X2), RP/UPLC-MS/MS negative mode, HILIC/UPLC-MS/MS negative mode, and backups. Samples were desolvated under nitrogen and stored at -80°C (for further details, see section 'Metabolomics sample processing' in Suppl. Materials)

CHROMATOGRAPHY PROTOCOL

- Protocol Title: Metabolon standard UPLC-MS metabolomics protocol
- Instrument: Waters ACQUITY Ultra-Performance Liquid Chromatography (UPLC) and Thermo Q-Exactive high resolution mass spectrometry (MS)
- Column: Waters UPLC BEH C18 column (2.1 x 100 mm, 1.7 µm) for RP/UPLC-MS/MS methods; Waters UPLC BEH Amide column (2.1 x 150 mm, 1.7 µm) for HILIC/UPLC-MS/MS
- Mobile phase: For RP, varied between water, methanol, acetonitrile, formic acid, perfluoropentanoic acid. For HILIC, water, acetonitrile with ammonium formate.
- Flow rate: varied between 350µL/min and 600 µL/min depending on the method
- Injection volume: 5 µL
- Run time: varied between ~3.5min and ~7minutes
- MS detection: Full scan at 35,000 resolution in positive and negative ionization modes

- Other details: 4 methods - 2 RP/UPLC-MS/MS positive, 1 RP/UPLC-MS/MS negative, 1 HILIC/UPLC-MS/MS negative (for further details, see section ‘Metabolomics sample processing’ in Suppl. Materials)

MASS SPECTROMETRY PROTOCOL

- Protocol Title: Metabolon standard metabolomics MS protocols
- Instrument: Thermo Q-Exactive high resolution MS
- Ionization: Heated electrospray ionization (HESI-II)
- Mass analyzers: Orbitrap mass analyzer
- Mass accuracy: < 5 ppm deviation
- Resolution: 35,000 for UPLC
- Scan speed: alternated between MS and data-dependent MS^n scans using dynamic exclusion
- Mass range: 70-1000 m/z
- Collision energy: Varied between methods
- Other details: Scan range, collision energy optimized for each method (for further details, see section ‘Metabolomics sample processing’ in Suppl. Materials)

DATA TRANSFORMATION PROTOCOL

- Protocol Title: Metabolon standard metabolomics data extraction and QC pipeline
- Software: Proprietary software by Metabolon
- Procedure: Peak detection, metabolite identification, raw data extraction, data normalization, imputation of missing values, log transformation, scaling
- Resulting data: Log-transformed, batch normalized and imputed peak area data
- Quality control: Median Relative Standard Deviation (RSD) of standards and

pooled samples monitored. Experimental samples randomized with quality control (QC) samples interspersed. Multiple replicates, internal standards, and total ion current normalization used.

- Other details: Raw data archived, extracted, and processed using Metabolon proprietary workflows (for further details, see section 'Metabolomics sample processing' in Suppl. Materials)

METABOLITE ANNOTATION PROTOCOL

- Protocol Title: Metabolon library annotation
- Database(s): Proprietary Metabolon reference library
- Identifiers: HMDB, PubChem, InChI, SMILES, CAS, ChemSpider, KEGG
- Annotation procedure: Automated comparison of ions, retention times and fragmentation signatures to library entries followed by manual curation
- Confidence scoring: Four confidence levels based on match to library entry
- Other details: for further details, see section 'Metabolomics sample processing' in Suppl. Materials

QUALITY CONTROL

Quality control (QC) samples and procedures: Pooled matrix samples, process blanks, solvent blanks, and internal standards tracked throughout experiments. Median RSD for standards and all endogenous metabolites monitored. Experimental samples randomized with QC samples spaced evenly (for further details, see section 'Metabolomics sample processing' in the Suppl. Materials)
