# Supplementary material for: Comprehensive blood metabolomics profiling of Parkinson’s disease reveals coordinated alterations in xanthine metabolism
Source: NPJ Parkinsons Dis. 2024 Mar 19;10:68. doi: 10.1038/s41531-024-00671-9 (PMC10951366; doi:10.1038/s41531-024-00671-9)
Supplement: Supplementary file 2 — Reporting Summary [file 41531_2024_671_MOESM2_ESM.pdf]

nature portfolio

Corresponding author(s): Enrico Glaab

Last updated by author(s): Nov 29, 2023

Reporting Summary

Nature Portfolio wishes to improve the reproducibility of the work that we publish. This form provides structure for consistency and transparency in reporting. For further information on Nature Portfolio policies, see our Editorial Policies and the Editorial Policy Checklist.

Statistics

For all statistical analyses, confirm that the following items are present in the figure legend, table legend, main text, or Methods section.

n/a	Confirmed	
☐	☒	The exact sample size (*n*) for each experimental group/condition, given as a discrete number and unit of measurement
☐	☒	A statement on whether measurements were taken from distinct samples or whether the same sample was measured repeatedly
☐	☒	The statistical test(s) used AND whether they are one- or two-sided<br>*Only common tests should be described solely by name; describe more complex techniques in the Methods section.*
☐	☒	A description of all covariates tested
☐	☒	A description of any assumptions or corrections, such as tests of normality and adjustment for multiple comparisons
☒	☐	A full description of the statistical parameters including central tendency (e.g. means) or other basic estimates (e.g. regression coefficient) AND variation (e.g. standard deviation) or associated estimates of uncertainty (e.g. confidence intervals)
☐	☒	For null hypothesis testing, the test statistic (e.g. *F*, *t*, *r*) with confidence intervals, effect sizes, degrees of freedom and *P* value noted<br>*Give P values as exact values whenever suitable.*
☒	☐	For Bayesian analysis, information on the choice of priors and Markov chain Monte Carlo settings
☒	☐	For hierarchical and complex designs, identification of the appropriate level for tests and full reporting of outcomes
☐	☒	Estimates of effect sizes (e.g. Cohen's *d*, Pearson's *r*), indicating how they were calculated

Our web collection on statistics for biologists contains articles on many of the points above.

Software and code

Policy information about availability of computer code

Data collection	No software was used for data collection
Data analysis	Differential abundance analyses were performed using the R software package limma (v3.52.2, RRID:SCR_010943). The R software package e1071 (https://cran.r-project.org/package=e1071, version 1.7-11) was used to train and apply the support vector machine models. Finally, pathway enrichment analyses for the metabolomics data were conducted using the MetaboAnalyst software (version 5.0, RRID:SCR_015539, DOI: 10.1093/nar/gkab382). The detailed use of these software packages is described in the Methods section of the manuscript.

For manuscripts utilizing custom algorithms or software that are central to the research but not yet described in published literature, software must be made available to editors and reviewers. We strongly encourage code deposition in a community repository (e.g. GitHub). See the Nature Portfolio guidelines for submitting code & software for further information.

Data

Policy information about availability of data

All manuscripts must include a data availability statement. This statement should provide the following information, where applicable:

- Accession codes, unique identifiers, or web links for publicly available datasets
- A description of any restrictions on data availability
- For clinical datasets or third party data, please ensure that the statement adheres to our policy

Public transcriptomics data was obtained from the GEO database (ID: GSE8397). Spreadsheet versions of the metabolite list and ranking tables which were too large

for inclusion into the Supplementary Materials document (Suppl. Tables 4-7, 11 and 12) have been made available on the public GitLab repository associated with the manuscript (https://gitlab.lcsb.uni.lu/bds/pd_metabolomics). The metabolomics dataset for this manuscript is not publicly available as it is linked to the Luxembourg Parkinson's Study and its internal regulations. Any requests for accessing the dataset can be directed to: request.ncer-pd@uni.lu.

Research involving human participants, their data, or biological material

Policy information about studies with human participants or human data. See also policy information about sex, gender (identity/presentation), and sexual orientation and race, ethnicity and racism.

Reporting on sex and gender

The term sex was used throughout the manuscript to refer to biological sex. We accounted for sex as a key variable influencing metabolite measurements by adjusting the statistical analyses for sex. We did not find any sex-specific changes in our analyses and therefore no sex-specific results are reported in our manuscript.

Reporting on race, ethnicity, or other socially relevant groupings

No socially relevant groupings were used in our analyses.

Population characteristics

The study cohort for the Parkinson's disease (PD) blood plasma metabolomics profiling consisted of 549 PD patients (including 56 de novo patients, who had not received dopaminergic medications) and 590 controls from the Luxembourg Parkinson's Study as part of the National Centre of Excellence in Research on Parkinson's disease (NCER-PD). Detailed population characteristics are already provided in Table 1 of the manuscript. In short, the covariate-relevant population characteristics include the following:

• Age range, mean and standard deviation for cases and controls: PD patients: 65.9 ± 10.7 years; de novo PD patients: 67.2 ± 11.4 years; Controls: 61.7 ± 11.7 years

• Sex distribution for cases and controls: PD patients - 188 female, 358 male; de novo PD patients - 14 female, 42 male; Controls - 206 female, 384 male

• MDS-UPDRS III: PD patients - 32.7 ± 14.5; de novo PD - 32.3 ± 14.2; Controls - 3.69 ± 5.0

• Hoehn & Yahr stage: PD patients - 2.1 ± 0.7; de novo PD - 1.8 ± 0.57

• Disease duration since initial symptom (years): PD patients - 12.3 ± 7.3; de novo PD - 6.68 ± 5.4

• Body Mass Index (kg/m2): PD patients - 27.5 ± 4.8; de novo PD - 28.2 ± 4.8; Controls - 27.6 ± 4.8

• Montreal Cognitive Assessment (MoCA): PD patients - 25.4 ± 3.2; de novo PD - 24.9 ± 3.3; Controls - 26.9 ± 2.6

• SCales for Outcomes in PArkinson's disease - Autonomic Dysfunction (SCOPA-AUT): PD patients - 14.4 ± 8.0; de novo PD - 8.9 ± 5.3; Controls - 7.3 ± 5.6

Recruitment

Participants in our metabolomics study were not recruited during this specific study, but were already part of the Luxembourg Parkinson's Study, a longitudinal observational study (see section "Study cohort" in the Methods section of the manuscript for more details). The biospecimens used for metabolomic profiling were previously collected from these participants. Although the study did not involve new recruitment, biases inherent in the original study design may still affect the results. Potential biases and limitations include:

1. Variation in dietary intake: The study did not control for the timing of the last meal or fasting status, which means that blood metabolite levels could vary with dietary intake. Therefore, potential systematic differences in diet between the study groups could lead to differences in metabolite profiles. For this reason, we have discussed potential dietary and gut microbiome influences in our interpretation of individual metabolite changes (see manuscript section "Significant metabolite changes in de novo Parkinson's disease and treated patients").

2. Diagnostic misclassification: Although the use of routine diagnostic imaging and annual follow-up increases diagnostic confidence in our study, a small proportion of misclassifications, particularly in the early stages of PD and in de novo patients, cannot be completely excluded. However, such misclassifications are not expected to significantly affect the qualitative results of our study, which consistently includes more than 50 subjects per group.

3. Sample handling and processing time: The time lag between blood collection and processing could introduce variability in the measurements, although there were no systematic differences in the handling of samples from different study groups.

4. Treatment effects: In PD patients receiving dopaminergic treatments, drug effects may confound metabolomic measurements. Therefore, we independently compared untreated de novo PD patients with controls to identify treatment-related changes in PD compared to controls.

5. Cognitive impairment: Group differences between patients and controls were found in cognitive impairment as assessed by the MoCA score. Although cognitive decline does not meet the definition of a confounder, if it was caused by the disease condition of interest, it could confound the results if it preceded the diagnosis of the disease.

A detailed discussion of these limitations can be found in the "Study limitations" section of the submitted manuscript.

Ethics oversight	The study was approved by the National Research Ethics Committee (CNER Ref: 201407/13) and the University of Luxembourg Ethics Review Panel (ref. ERP 18-042).

Note that full information on the approval of the study protocol must also be provided in the manuscript.

Field-specific reporting

Please select the one below that is the best fit for your research. If you are not sure, read the appropriate sections before making your selection.

☒ Life sciences ☐ Behavioural & social sciences ☐ Ecological, evolutionary & environmental sciences

For a reference copy of the document with all sections, see nature.com/documents/nr-reporting-summary-flat.pdf

Life sciences study design

All studies must disclose on these points even when the disclosure is negative.

Sample size	As this was a cohort-wide analysis, covering all relevant available biosamples in the Luxembourg Parkinson Study, no sample size calculation was performed (regarding the number of samples per group, see the section on 'Population characteristics above').
Data exclusions	No data samples were excluded from the analysis
Replication	This was a cohort-wide discovery analysis without an external replication cohort. Therefore, in the "Study limitations" section of our manuscript, we state that "for both the optimization and robust validation of metabolite-derived machine learning models, independent analyses with larger sample sizes of de novo patients across multiple distinct cohorts are needed".
Randomization	Our study involved only case-control comparisons (comparing all cases against all controls) and correlative analyses for all cases; therefore no sub-group assignments involving randomization were required in this study.
Blinding	This study focused primarily on discovery analyses using case-control comparisons, which inherently do not require subgroup allocations or blinding. Furthermore, the objective nature of the measured metabolomic data and the statistical methods used for analysis significantly reduced the risk of any subjective bias influencing the results.

Reporting for specific materials, systems and methods

We require information from authors about some types of materials, experimental systems and methods used in many studies. Here, indicate whether each material, system or method listed is relevant to your study. If you are not sure if a list item applies to your research, read the appropriate section before selecting a response.

Materials & experimental systems

n/a	Involved in the study	
☒	☐	Antibodies
☒	☐	Eukaryotic cell lines
☒	☐	Palaeontology and archaeology
☒	☐	Animals and other organisms
☒	☐	Clinical data
☒	☐	Dual use research of concern
☒	☐	Plants

Methods

n/a	Involved in the study	
☒	☐	ChIP-seq
☒	☐	Flow cytometry
☒	☐	MRI-based neuroimaging

Plants

Seed stocks	*Report on the source of all seed stocks or other plant material used. If applicable, state the seed stock centre and catalogue number. If plant specimens were collected from the field, describe the collection location, date and sampling procedures.*
Novel plant genotypes	*Describe the methods by which all novel plant genotypes were produced. This includes those generated by transgenic approaches, gene editing, chemical/radiation-based mutagenesis and hybridization. For transgenic lines, describe the transformation method, the number of independent lines analyzed and the generation upon which experiments were performed. For gene-edited lines, describe the editor used, the endogenous sequence targeted for editing, the targeting guide RNA sequence (if applicable) and how the editor was applied.*
Authentication	*Describe any authentication procedures for each seed stock used or novel genotype generated. Describe any experiments used to assess the effect of a mutation and, where applicable, how potential secondary effects (e.g. second site T-DNA insertions, mosiacism, off-target gene editing) were examined.*
